# Validation of a hypoxia related gene signature in multiple soft tissue sarcoma cohorts

**DOI:** 10.18632/oncotarget.23280

**Published:** 2017-12-12

**Authors:** Lingjian Yang, Laura Forker, Joely J. Irlam, Nischalan Pillay, Ananya Choudhury, Catharine M. L. West

**Affiliations:** ^1^ Translational Radiobiology Group, Division of Cancer Sciences, University of Manchester, Manchester Academic Health Science Centre, Christie Hospital, Manchester, UK; ^2^ Cancer Institute, University College London, London, UK; ^3^ Histopathology, Royal National Orthopaedic Hospital, Stanmore, UK; ^4^ NIHR Manchester Biomedical Research Centre, Central Manchester University Hospitals NHS Foundation Trust, Manchester Academic Health Science Centre, Manchester, UK

**Keywords:** soft tissue sarcoma, tumor hypoxia, gene expression signature, prognostic biomarker

## Abstract

**Purpose:**

There is a need for adjuvant/neo-adjuvant treatment strategies to prevent metastatic relapse in soft tissue sarcoma (STS). Tumor hypoxia is associated with a high-risk of metastasis and is potentially targetable. This study aimed to derive and validate a hypoxia mRNA signature for STS for future biomarker-driven trials of hypoxia targeted therapy.

**Materials and Methods:**

RNA sequencing was used to identify seed genes induced by hypoxia in seven STS cell lines. Primary tumors in a training cohort (French training) were clustered into two phenotypes by seed gene expression and a *de novo* hypoxia signature derived. Prognostic significance of the *de novo* signature was evaluated in the training and two independent validation (French validation and The Cancer Genome Atlas) cohorts.

**Results:**

37 genes were up-regulated by hypoxia in all seven cell lines, and a 24-gene signature was derived. The high-hypoxia phenotype defined by the signature was enriched for well-established hypoxia genes reported in the literature. The signature was prognostic in univariable analysis, and in multivariable analysis in the training (*n* = 183, HR 2.16, *P* = 0.0054) and two independent validation (*n* = 127, HR 3.06, *P* = 0.0019; *n* = 258, HR 2.05, *P* = 0.0098) cohorts. Combining information from the *de novo* hypoxia signature and a genome instability signature significantly improved prognostication. Transcriptomic analyses showed high-hypoxia tumors had more genome instability and lower immune scores.

**Conclusions:**

A 24-gene STS-specific hypoxia signature may be useful for prognostication and identifying patients for hypoxia-targeted therapy in clinical trials.

## INTRODUCTION

Soft tissue sarcomas (STS) are a group of rare cancers arising from mesenchymal cells that account for less than 1% of solid tumors in adults. They are extremely heterogeneous with over 50 malignant histologic subtypes and can occur in any anatomical position [1]. Most patients present with localized disease, for which surgery (+/– radiotherapy) is the cornerstone of treatment. Despite high local control rates, the five-year overall survival is only around 50% in patients with high-risk disease (high grade, deep, large tumors) [2, 3], where most deaths are attributable to distant metastasis. Clinical trials have failed to demonstrate a consistent overall survival benefit for conventional neo-adjuvant/adjuvant chemotherapy, which is not currently recommended as an international standard of care [4]. Novel strategies to target and prevent metastatic spread are urgently needed.

Failure of previous neo-adjuvant/adjuvant trials on unselected patients may reflect the molecular heterogeneity of STS. Assigning neo-adjuvant chemotherapy based on histologic subtype has also been unsuccessful [5]. An alternative approach may be to target a feature of tumor biology present across multiple subtypes and associated with an increased risk of metastasis.

Hypoxia is a generic feature of the tumor microenvironment which can drive metastatic spread [6] and is associated with distant relapse in localized STS [7]. Systemic agents targeting hypoxic cell populations [8] reduced the risk of lung metastasis in animal sarcoma models when administered adjuvantly [9, 10]. A biomarker of tumor hypoxia could identify patients for hypoxia targeted therapy in STS in clinical trials.

Hypoxia mRNA signatures are progressing towards clinical implementation as predictive biomarkers for response to hypoxia-targeted therapies. A 26-gene hypoxia signature is currently undergoing prospective validation in the UK phase III NIMRAD trial in head and neck cancer [11]. The transcriptomic response to hypoxia varies by tumor type and hypoxia signatures perform better when they are tumor type specific [12]. No STS-specific hypoxia signature exists, although a head and neck hypoxia signature had prognostic significance in a small cohort of high-grade STS patients [13]. The main aim of this study was to derive an STS-specific hypoxia mRNA signature by generating new *in vitro* data and accessing publically available *in vivo* whole transciptome expression data, and to test its prognostic significance in multiple clinical cohorts.

## RESULTS

### Transcriptomic response to hypoxia is preserved across seven STS cell lines

RNA sequencing data were generated in triplicate for seven cell lines, representing the most common histologic STS subtypes reported in adults. A replicate for HT1080 and one for SW872 had lower percentages of reads counted into genes (∼50%) compared to the other samples ( > 90%). The two replicates were outliers in principal component analysis and were therefore omitted from further analysis. 734, 451, 553, 875, 1367, 1160 and 966 genes were up-regulated by hypoxia in 93T449, HT1080, SKUT1, SNF96-2, SW684, SW872 and SW982, respectively. Monte Carlo sampling (*n* = 1000) generating seven random gene sets showed an average of 5.7 genes appeared in ≥ 3 gene sets by chance. By contrast, 37 genes were induced by hypoxia in all seven STS lines (Supplementary Table 2). 93, 179 and 342 genes were significantly up-regulated by hypoxia in more than six, five and four cell lines, respectively. The number of genes down-regulated by hypoxia was smaller with 5, 24, 58 and 187 genes suppressed in all seven, six, five and four cell lines, respectively. Simulation of random gene sets showed only 1.7 genes would appear in more than three cell lines by chance. The results indicated that transcriptomic response to hypoxia was preserved across molecularly heterogeneous STS cell lines.

Of the genes induced by hypoxia in all seven cell lines, *SLC2A1, BNIP3L, BNIP3, MXI1, PDK1, P4HA2, DDIT4, SLC2A1* were among the 20 genes most frequently included in published hypoxia signatures [12]. *CA9*, a well-studied hypoxia target gene, was up-regulated by hypoxia in five STS cell lines. Gene ontology terms enriched with genes inducible by hypoxia ≥ 5 cell lines include response to hypoxia, canonical glycolysis, positive regulation of apoptotic process, oxidation-reduction process, and circadian regulation of gene expression. Enriched KEGG pathways included HIF-1 signaling pathway, glycolysis/gluconeogenesis, biosynthesis of antibiotics and carbon metabolism (Supplementary Tables 3 and 4). For many of the above identified processes, their alteration under hypoxia was previously documented in other tumor sites [14–16]. The 187 genes down-regulated in ≥ 4 cell lines were mainly enriched in G1/S cell cycle, nucleolus, DNA replication and RNA binding processes (Supplementary Table 5).

### Genes induced *in vitro* segregate STS *in vivo* into high- and low-hypoxia phenotypes

The 33 protein coding genes induced by hypoxia in all seven cell lines and available in both the French and TCGA cohorts were used as seed genes to derive a hypoxia signature *in vivo*. In the French training cohort, 182 STS clustered into two groups (*n* = 48 and *n* = 134) based on the expression similarity of the 33 seed genes. 193 genes (1.2% of all genes) were significantly up-regulated in the 48-tumor group (multiple test corrected fdr < 0.05 and > 1.5 fold change). Twenty-four of the 33 seed genes (73%) were *significantly* up-regulated in the 48-tumor group. Another eight seed genes were up-regulated but not significantly and one seed gene was slightly down-regulated. Of those not in the seed gene list, the well-recognized hypoxia-inducible *CA9, CA12* and *ADM* were the 2nd, 6th and 18th most up-regulated genes (by fold change) in the 48-tumor group. Other *in vitro* induced hypoxia genes were also highly expressed in the 48-tumor group: 45%, 38% and 25% of genes up-regulated by hypoxia in six, five and four cell lines, respectively. In comparison only 1.2% of the whole ∼18,000 genes studied were up-regulated in the 48-tumor group. Gene set enrichment analysis identified 168 pathways significantly enriched in the 48-tumor group (fdr < 0.01), 16 of which were hypoxia gene sets derived in different tumor sites and experimental conditions (Supplementary Table S6). The analyses therefore clearly indicate that the two tumor groups have distinct high-hypoxia and low-hypoxia molecular phenotypes.

### A *de novo* 24-gene hypoxia signature is derived using the *in vitro* induced genes as seeds

**Figure 1:**
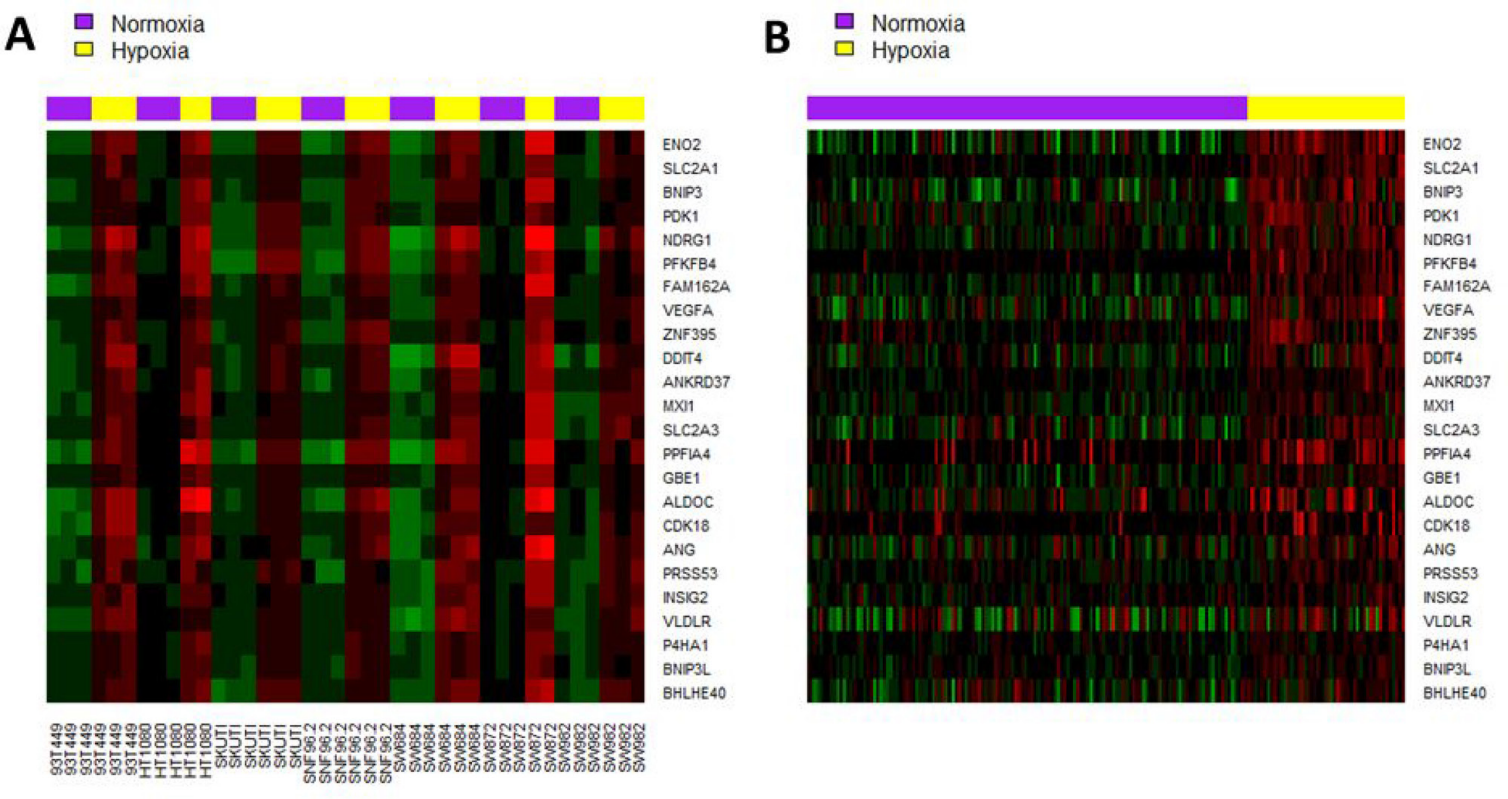
Heatmap of the *de novo* 24-gene hypoxia signature in (**A**) seven STS cell lines under hypoxia (1% oxygen, 24 hours) and normoxia conditions; (**B**) French training cohort. Tumor samples were clustered into high-hypoxia and low-hypoxia phenotypes based on the expression pattern of the 24 signature genes.

A *de novo* 24-gene hypoxia signature was derived from the 33 seed genes using prediction analysis for microarray (PAM) [17], which had an optimal 10-fold cross validation accuracy of 96.7. The 24 genes are listed in Supplementary Table 7. Expression of the 24 signature genes in seven cell lines and in the French training cohort are illustrated in Figure 1A and 1B, respectively. In the French validation cohort, the *de novo* 24-gene hypoxia signature assigned 27 (21%) and 100 (79%) of tumors into high-hypoxia and low-hypoxia groups respectively. In the TCGA cohort, the *de novo* signature assigned 51 (19%) and 212 (81%) tumors into high-hypoxia and low-hypoxia groups respectively (Supplementary Table S8).

### Expression of the *de novo* signature correlates with another measure of hypoxia

As no cohorts were available, it was not possible to investigate relationships between our *de novo* signature with other measures of hypoxia. The most commonly used approach for assessing hypoxia in tumors involves measuring the protein expression of strongly inducible hypoxia genes, the most widely studied being CA9, GLUT1 (*SLC2A1*) and HIF1α. Data were available from another project where both mRNA and protein expression of *CA9*, *GLUT1* (*SLC2A1*) and *HIF1a* were studied in a cohort of urothelial cancer patients [18]. Significant correlations were seen for *CA9* (r = 0.47) and *GLUT1* (*SLC2A1,* r = 0.56), but not *HIF1a* (r = 0.10, Supplementary Figure 2). As there are correlations between mRNA and protein expression for the widely studied hypoxia markers, we investigated them further to provide evidence the 24-gene signature would select tumors identified as hypoxic using another approach. In three STS cohorts, mRNA expression of *CA9, SLC2A1* and *HIF1a* was significantly higher in tumors stratified as high-hypoxia by the 24-gene signature (Supplementary Figure 3).

### Relationship of the *de novo* hypoxia signature with clinico-pathological factors

There was an association between the 24-gene hypoxia signature and histological subtype (chi-square test *P* = 0.03 for combined French cohort and 0.004 for TCGA). 29% of leiomyosarcoma tumors were stratified as high-hypoxia by the *de novo* 24-gene signature in comparison with a level of ∼11% for liposarcoma and ∼20% across all subtypes. There were no associations between the hypoxia signature with tissue site, age at diagnosis, gender and pathological lesion length (Supplementary Table 9).

### High-hypoxia tumors have a poor prognosis

**Figure 2:**
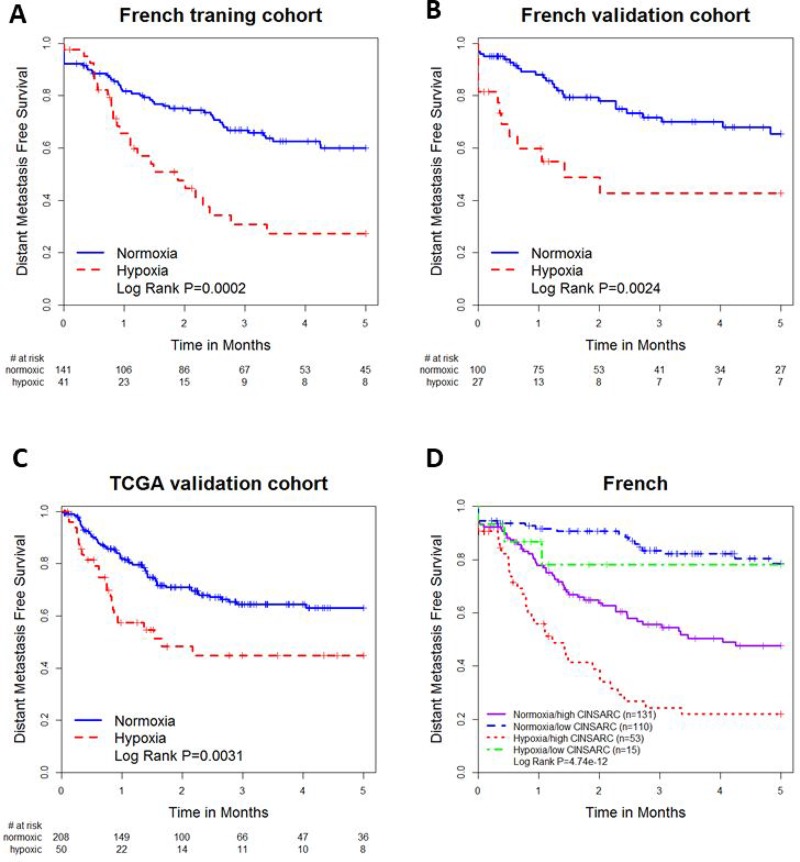
Kaplan-Meier plots for (**A**) the French training cohort stratified by the 24-gene signature; (**B)** the French validation cohort stratified by the 24-gene hypoxia signature; (**C**) the TCGA cohort stratified by the 24-gene hypoxia signature; (**D**) the combined French cohort stratified by both hypoxia signature and CINSARC signature.

In the French training cohort, tumors stratified by the *de novo* 24-gene signature as high-hypoxia had significantly worse 5-year DMFS than those stratified as low-hypoxia (HR 2.43, 95% CI 1.49-3.96, *P* = 0.00036, Figure 2A). Tumors stratified as high-hypoxia also had poorer DMFS in the French (HR 2.73, 95% CI 1.40–5.34, *P* = 0.0033, Figure 2B) and TCGA (HR 2.03, 95% CI 1.26–3.29, *P* = 0.0037, Figure 2C) validation cohorts. Prognostic significance was retained in multivariable analyses adjusting for histological diagnosis, tumor tissue site, gender, age and pathological lesion length in the French training (HR 2.16, 95% CI 1.25–3.70, *P* = 0.0054), French validation (HR 3.06, 95% CI 1.51–6.19, *P* = 0.0019) and TCGA (HR 2.05, 95% CI 1.19–3.53, *P* = 0.0098) cohorts (Table 1).

**Table 1:** Univariable and multivariable analysis of the *de novo* 24-gene hypoxia signature

Study	Variable	Univariable Analysis		Mulitvariable Analysis	
Cohort studies		HR (95% CI)	*P*-value	HR (95% CI)	*P*-value
**French training**	**Hypoxia signature**	2.43 (1.49-3.96)	0.00036	2.16 (1.25–3.70)	0.0054
	**Tumor tissue site**				
	Extremities				
	Head and neck	0	1	0	1
	Internal trunk	1.14 (0.64–2.03)	0.65	1.26 (0.66–2.40)	0.49
	Trunk wall	1.62 (0.88–2.96)	0.12	1.34 (0.72–2.49)	0.35
	**Histologic diagnosis**				
	Liposarcoma				
	Undifferentiated sarcoma	1.19 (0.60–2.37)	0.62	1.30 (0.60–2.79)	0.51
	Leiomyosarcoma	2.23 (1.14–4.37)	0.02	1.91 (0.94–3.88)	0.076
	Other	1.48 (0.58–3.75)	0.41	1.12 (0.39–3.16)	0.84
**French validation**	**Hypoxia signature**	2.73 (1.40–5.34)	0.0033	3.06 (1.51–6.19)	0.0019
	**Tumor tissue site**				
	Extremities				
	Head and neck	0	1	0	1
	Internal trunk	1.07 (0.49–2.32)	0.87	1.70 (0.70–4.15)	0.24
	Trunk wall	1.12 (0.50–2.52)	0.78	1.02 (0.43–2.40)	0.97
	**Histologic diagnosis**				
	Liposarcoma				
	Undifferentiated sarcoma	2.81 (0.65–12.18)	0.17	3.86 (0.78–19.10)	0.10
	Leiomyosarcoma	7.44 (1.73–32.00)	0.0071	9.83 (2.12–45.54)	0.003
	Other	0	1	1.14 (0.1–13.04)	0.92
**TCGA validation**	**Hypoxia signature**	2.03 (1.26–3.29)	0.0037	2.05 (1.19–3.53)	0.0098
	**Tumor tissue site**				
	Extremity				
	Abdomen	0.42 (0.17–1.08)	0.071	0.39 (0.13–1.19)	0.099
	Head and neck	0	1	0	1
	Pelvic	0.58 (0.21–1.64)	0.31	0.63 (0.19–2.14)	0.46
	Retroperitoneal	0.61 (0.36–1.05)	0.074	0.88 (0.45–1.71)	0.71
	Thorax	0.44 (0.17–1.13)	0.088	0.50 (0.19–1.30)	0.16
	Uterine	1.17 (0.62–2.19)	0.64	1.13 (0.49–2.63)	0.77
	**Histologic diagnosis**				
	Liposarcoma				
	Leiomyosarcoma	3.33 (1.63–6.81)	0.00099	3.82 (1.63–8.97)	0.0021
	Malignant Peripheral Nerve Sheath Tumors	1.74 (0.37–8.03)	0.48	2.47 (0.44–13.88)	0.30
	Myxofibrosarcoma	2.70 (1.10–6.64)	0.031	3.26 (1.09–9.72)	0.034
	Undifferentiated pleomorphic sarcoma	2.70 (1.19–6.11)	0.017	3.67 (1.34–10.05)	0.012
	Synovial	1.91 (0.52–7.06)	0.33	3.14 (0.67–14.75)	0.15
	**Male**	0.98 (0.64–1.50)	0.91	1.53 (0.94–2.51)	0.088
	**Pathological lesion length**	1.00 (0.98–10.2)	0.89	1.02 (0.99–1.04)	0.064
	**Age**	0.99 (0.98–1.01)	0.69	0.99 (0.97–1.01)	0.44

The French sarcoma group [19] developed a highly prognostic 67-gene CINSARC (Complexity INdex in SARComas) transcriptomic signature. Combining the 24-gene hypoxia and the CINSARC signatures improved prognostication (log rank *P* = 4.74*10^-12^, Figure 2D) compared with the hypoxia (*P* = 1.6*10^-6^) or CINSARC (*P* = 1.47*10^-9^) signatures alone. Using patients stratified as low-hypoxia and low CINSARC as a reference, patients stratified as high-hypoxia and high CINSARC had a significantly poorer outcome (HR 6.74, 95% CI 3.84–11.84, *P* = 3.13^*^10^-11^). Patients with low-hypoxia signature and high CINSARC signature also had a poor prognosis (HR 3.18, 95% CI 1.89–5.37, *P* = 1.44^*^10^-5^).

### Comparison with a published hypoxia signature

A published 15-gene hypoxia signature for head and neck cancer had prognostic value in high-grade STS [13]. In two out of the three STS cohorts, the 15-gene signature also achieved prognostic significance in multivariable analyses (French training: HR 1.8, *P* = 0.017; French validation: HR 1.51, *P* = 0.21; TCGA: HR 1.72, *P* = 0.03). Due to the higher HRs in multivariable analyses and significance across all three cohorts, the *de novo* 24-gene signature is a better prognostic biomarker in STS.

### High hypoxia tumors have high genomic instability

Hypoxia was recently proposed as a driver of genome instability [20]. Therefore we investigated the association between the 24-gene hypoxia signature with two indices reflecting genome instability. In the TCGA cohort, data are available for the percentage of the genome with copy number alterations. Genome alterations were significantly higher in high- versus low-hypoxia tumors (47% *vs* 33%; *t*-test *P* = 0.0007, Supplementary Figure 4). The CINSARC signature was used as a measure of genome instability in the combined French cohort. There were more high-risk CINSARC classifications in high- versus low-hypoxia tumors (76% *vs* 48%; chi-square test *P* = 0.0035). Similarly, there were more high-hypoxia classifications in high versus low CINSARC signature tumors (31% *vs* 12%; Supplementary Table 10). Therefore, our data are consistent with hypoxia being a driver of genome complexity.

### High hypoxia tumors have low immune cell infiltration

The ESTIMATE [21] computational algorithm was used to evaluate the presence of tumor-infiltrating immune cells. Of 30 tumor types included in TCGA, STS had the 9^th^ highest abundance and the largest intra-cancer variability in estimated immune cell infiltration (Supplementary Figure 5). High-hypoxia tumors had significantly lower immune infiltration scores than the low-hypoxia tumors (*t*-test *P* = 0.0007, Supplementary Figure S6).

In the French training cohort, Gene set enrichment analysis showed that 19 of the 49 gene ontology terms that were significantly (fdr < 0.01) over-represented in the low-hypoxia tumors were related to innate and adaptive immune response, T cell proliferation, inflammatory response, B cell and lymphocyte mediated immunity. Another three terms relating to interferon signaling were also enriched. Similar results were seen for KEGG pathways with eight immune and ten interferon signaling pathways significantly up-regulated in the low-hypoxia tumors (Supplementary Table 11). Interferon signaling proteins have important functions in initializing immune response and activing immune cells [22]. We also analyzed the associations between hypoxia signature status and expression of 192 cancer-related proteins available for the TCGA cohort. Three of the ten protein markers most down-regulated in high- versus low-hypoxia tumors induce immune responses (CD31, Lck and PD1; fdr < 0.05; Supplementary Figure 7). Our analyses, therefore, suggest that in STS hypoxia is associated with an impaired immune response.

## DISCUSSION

Tumor hypoxia is associated with metastatic relapse in STS and can be targeted pharmacologically. Despite showing early promise, the bioreductive drug TH-302 did not improve survival in a phase III trial in unselected patients with metastatic STS [23], highlighting the need for carefully designed biomarker driven trials. There is currently no reliable hypoxia biomarker available for clinical trials. Direct measurements using oxygen electrodes are invasive and impractical [24], but a biomarker that can be measured using pre-treatment diagnostic biopsies would be attractive. Immunohistochemical expression of widely studied hypoxia markers (CA9, GLUT1, HIF1a) has been explored but findings are equivocal [25–27]. A limitation of immunohistochemistry is the need for manual pathology scoring and concern over using a single protein marker in histologically heterogeneous STS. A transcriptomic signature overcomes these issues. This study generated a novel 24-gene signature that reflects tumor hypoxia in STS and was validated as a prognostic marker in two independent cohorts.

To be useful as a predictive biomarker the signature must reflect hypoxia in STS. It was shown previously that the transcriptional response to hypoxia varies across different cancer types [12,28], which highlights the need for tumor type specific signatures. A hypoxia signature originally derived for head and neck cancer was shown to be prognostic in a small cohort of soft tissue sarcomas [13], but correlated poorly with oxygen electrode measurements in 16 patients. In addition, our *de novo* 24-gene signature performed better than the head and neck cancer classifier across the three cohorts studied.

In order to ensure our signature reflects hypoxia in STS, our study was the first to assess comprehensively the transcriptome-wide response to hypoxia in STS cell lines. Despite the molecular heterogeneity of STS, the transcriptomic response to hypoxia was well preserved across the seven cell lines, implying that a single signature could be applied to all adult STS subtypes in clinical practice. Among the genes consistently up-regulated by hypoxia, many were well-known hypoxia genes that are included in published signatures. The genes induced by hypoxia in all seven STS cell lines were used to define a high-hypoxia and a low-hypoxia phenotype *in vivo.* Hypoxia genes and pathways identified in different tumor sites and experimental conditions were highly enriched in the high-hypoxia phenotype, increasing confidence that the signature reflects hypoxia in STS.

Genes induced by hypoxia *in vitro* were used as seed genes to derive a STS-specific 24-gene signature. The *de novo* hypoxia signature had strong and independent prognostic value in three STS cohorts and was validated across two different platforms (array versus RNA sequencing). In current practice the strongest prognostic indicator for STS is grade [4]. The main limitation of this work is the omission of grade from the multivariate analysis. Further development of the signature for clinical use will involve two new cohorts with extensive clinical data available (including the UK phase III VorteX trial cohort [29]) to allow assessment of independence from grade. Another proposed prognostic marker for STS is the 67 gene CINSARC signature [19]. Combining the CINSARC and hypoxia signatures improved prognostication and may be helpful in identifying very high-risk patients.

*In vitro* studies suggest that tumor hypoxia can drive genome instability by altering DNA damage response and producing free radical damage [20], and so promote tumor aggressiveness. High-hypoxia tumors (determined by the signature) had high levels of somatic copy number alterations, which is a surrogate marker of genome instability. Our study is the first to explore relationships between hypoxia and genome instability in primary STS. Future work could investigate drugs targeting aberrant DNA repair mechanisms in high-hypoxia STS.

There is increasing interest in understanding how reduced immune responses promote cancer progression and can be targeted with drugs such as immune checkpoint inhibitors STS [30]. STS had the largest variation in immune infiltrates (measured by ESTIMATE) of all the cancer types included in TCGA. High-hypoxia STS had significantly lower immune scores (reflecting decreased immune infiltration) compared to low-hypoxia STS. This could be clinically useful when identifying patients for immunotherapy trials in STS as patients with high-hypoxia tumors may not benefit. Further investigation of the relationship between hypoxia and immune response in STS is warranted.

In summary, we generated and validated the first STS specific hypoxia signature. The signature could be used as a biomarker to select enriched populations for hypoxia targeted therapy in STS in future trials.

## MATERIALS AND METHODS

### Methodology and study design

We previously derived hypoxia mRNA signatures for head and neck squamous cell [31] and urothelial [18] cancer using literature curated hypoxia-inducible genes as seeds. The seed genes used however could be: 1) non-specific for STS as transcriptomic response to hypoxia is tumor site specific; 2) of low quality as they were identified from different labs using heterogeneous experimental conditions (*in vitro*/*in vivo*/bioinformatics analysis) and technologies. The updated methodology used in this study involved generating seed genes that are consistently up-regulated by hypoxia in multiple STS cell lines. Our previous hypoxia signatures assigned continuous signature scores to individual tumors, and high-hypoxia and low-hypoxia groups were created by splitting from manually specified cut-off values (e.g. cohort median signature score). Our updated methodology applied a machine learning method in a training cohort to partition tumors into two groups (high-hypoxia and low-hypoxia) based on the maximum inter-group difference in gene expression of seed genes. A *de novo* signature was then derived with cross validation that was able to stratify new tumors into high-hypoxia and low-hypoxia groups without the need for a manual cut-off. A schematic presentation of the study design is provided in Supplementary Figure 1.

### Cell culture

HT1080, SKUT1, sNF96.2, 93T449, SW684, SW872 and SW982 were purchased from the American Type Culture Collection (ATCC, Teddington, Middlesex, UK), and cultured according to their recommendations. Cell lines and culture conditions are summarized in Supplementary Table 1. Cell lines were authenticated by the Promega Powerplex 21 System and underwent mycoplasma screening (Molecular Biology Core Facility, CRUK Manchester Institute, UK).

### Hypoxia exposure

Cells were seeded in 75 cm^2^ flasks at an appropriate density to achieve 60% confluence after 48 hours culture under normoxia for each individual cell line (calculated from growth curves, data not shown). Cells were cultured under normoxia for 24 hours, after which the media was changed prior to a further 24 hours culture under normoxia or 1% oxygen (Ruskin Invivo2 400 hypoxia workstation, Ruskinn Technology Ltd, Bridgend, UK). 1% oxygen was chosen as it is widely used in the literature and HIF, a major regulator of transcriptional responses to hypoxia, expression stabilizes in cell lines at this oxygen concentration. Experiments were repeated for three different passages for each cell line. Hypoxia exposed cells were harvested under 1% hypoxia.

### RNA extraction and sequencing

RNA was extracted using the Phenol-Choroform (TRIZOL) method and cleaned using a Qiagen RNeasy mini kit (cat no 74104). Sample yields were obtained using a Nanodrop spectrophotometer (ThermoFisher Scientific) and RNA integrity (RIN) measured using an Agilant Bioanalyzer. The 42 samples had RIN values between 8.6 and 10 and were sent for RNA sequencing. The sequencing libraries were prepared using Illumina’s TruSeq stranded mRNA protocol and sequenced using the Illumina HiSeq4000 platform (150 cycle protocol - 2x76bp paired end reads) at a depth of ∼50 M (pairs of) reads per sample.

### Identification of genes induced by hypoxia *in vitro*

Unmapped paired-end sequences from an Illumina HiSeq4000 sequencer were tested by FastQC v0.11.5 using a variety of metrics. Sequence adapters were removed and reads trimmed using Trimmomatic (v0.36) [32]. The reads were mapped against the reference human genome (version hg38) using STAR (v2.4.2) [33]. Counts per gene were calculated with HTSeq (v0.6.1) [34] using annotation from GENCODE (v24). Normalization and differential expression were calculated with DESeq2 (v1.10.1) [35]. Genes up- and down-regulated by hypoxia were identified with DESeq2 (multiple test corrected fdr < 0.05 and ≥ 2 fold change on pre-log2 transformed expression). Gene ontology terms and KEGG pathways enriched with protein-coding genes consistently induced by hypoxia in multiple cell lines were identified using DAVID v6.8 [36] (Benjamini corrected *P* < 0.1). RNA seq data were uploaded to ArrayExpress (accession E-MTAB-6087).

### STS patient cohort and expression normalization

Two independent patient cohorts with whole transcriptome and clinical outcome data were curated from the French sarcoma group [19] (GEO accession: GSE21050) and the cancer genome atlas project (TCGA) [37]. In the French cohort, gene expression data were generated for 310 fresh frozen patient samples on Affymetrix U133 plus2 arrays. In the TCGA cohort, 246 patient tumors were surgically resected and RNA sequencing data were generated from fresh frozen material. The French cohort was divided into training (*n* = 183) and validation (*n* = 127) cohorts as per the original publication.

For the French cohort, raw CEL files were downloaded and normalized with the GC-RMA method [38]. When multiple probe sets map to the same gene, their median expressions were used. For TCGA cohort, “rsem.genes.results” files from Broad institute Firehose were downloaded and transferred to transcript per million (TPM). A small positive constant of 0.25 was added to TPM before log2 transformation for gene expression. Only the genes present in both patient cohorts were analyzed. Percentage of copy number alteration for TCGA tumors were obtained from cBioPortal [39]. The Cancer Proteome Atlas (TCPA) project measured expression of ∼200 cancer-related proteins covering major signaling pathways using a high-throughput reverse phase protein array [40]. These protein expression data for the TCGA STS were downloaded from firehose.

### Development and validation of a hypoxia signature

Genes induced by hypoxia in seven STS cell lines served as seeds. In the French training cohort, tumors were clustered into high-hypoxia and low-hypoxia groups using *K*-means clustering in R (v3.2.3; 1000 random starts) on log2 transformed and median centered expression of the seed genes. Differential expression analysis was performed with LIMMA [41] (v3.26.9). A signature was derived using prediction analysis for microarrays (PAM) [17], a classification model that simultaneously performs gene selection, and the seed genes as candidates. 10-fold cross validation was performed and the signature with the lowest classification errors was selected as the final *de novo* signature. PAM also generates class centroids for each signature gene. Given a new tumor sample, the *de novo* signature compares its gene expression to high-hypoxia and low-hypoxia class centroids. The class whose centroid that it is closest to, in squared distance, is the predicted class for that new sample.

### Endpoint and statistical analysis

The clinical endpoint used was distant metastasis free survival (DMFS) as specified in the original publications for the different cohorts. All patients were censored at 5 years. The chi-square test was used to compare proportions across categorical factors. The Welch *t* test was used to compare mean values for continuous variables between two groups. Survival estimates were performed using the Kaplan-Meier method and differences compared using the log-rank test. Hazard ratios (HR) and 95% confidence intervals (CI) were obtained using Cox proportional hazard model. Multivariable analyses were performed adjusting the *de novo* hypoxia signature for histologic subtypes, age, gender, tumor site and pathological lesion length. Association with the CINSARC signature [19], an index of genomic instability, was evaluated using Kaplan-Meier survival estimates and a Cox proportional hazards model. All *P*-values were two sided and statistical significance was set as 0.05.

### Comparison with a literature hypoxia signature

We also evaluated a 15-gene hypoxia signature developed for head and neck cancer [13]. Median expressions of the 15 genes were taken as signature scores. Tumors were split into two groups using cohort median, upper and lower quartile values. Survival analyses were performed as described above. Results were broadly similar across the three cut-offs and therefore only those for median cut-off were presented.

## SUPPLEMENTARY MATERIALS FIGURES AND TABLES








