# Correction: Validation of a hypoxia related gene signature in multiple soft tissue sarcoma cohorts

**DOI:** 10.18632/oncotarget.26783

**Published:** 2019-03-08

**Authors:** Lingjian Yang, Laura Forker, Joely J. Irlam, Nischalan Pillay, Ananya Choudhury, Catharine M.L. West

**Affiliations:** ^1^ Translational Radiobiology Group, Division of Cancer Sciences, University of Manchester, Manchester Academic Health Science Centre, Christie Hospital, Manchester, UK; ^2^ Cancer Institute, University College London, London, UK; ^3^ Histopathology, Royal National Orthopaedic Hospital, Stanmore, UK; ^4^ NIHR Manchester Biomedical Research Centre, Central Manchester University Hospitals NHS Foundation Trust, Manchester Academic Health Science Centre, Manchester, UK

**This article has been corrected:** The correct Acknowledgments information is given below:

**ACKNOWLEDGMENTS**

We thank Leo Zeef and Andy Hayes of the Bioinformatics and Genomic Technologies Core Facilities at the University of Manchester for providing support with regard to RNAseq. This work was funded by a Cancer Research UK Major Centre award [C147/A18083]; Sarcoma UK (SUK205.2016); and the NIHR Manchester Biomedical Research Centre. The results here are partly based upon data generated by the TCGA Research Network: http://cancergenome.nih.gov/.

Original article: Oncotarget. 2018; 9:3946-3955. https://doi.org/10.18632/oncotarget.23280

