# Supplementary material for: Validation of a hypoxia related gene signature in multiple soft tissue sarcoma cohorts
Source: Oncotarget. 2017 Dec 12;9(3):3946–55. doi: 10.18632/oncotarget.23280 (PMC5790513; doi:10.18632/oncotarget.23280)
Supplement: Supplementary file 1 [file oncotarget-09-3946-s001.pdf]

Validation of a hypoxia related gene signature in multiple soft tissue sarcoma cohorts

SUPPLEMENTARY MATERIALS

Supplementary Table 1: Soft tissue sarcoma cell lines used in this work

Cell Line	Code	Subtype	Medium	FBS	Hepes	Incubator Conditions	Subcultivation Ratio
HT1080	CCL-121	Fibrosarcoma	eagle's minimum essential (Glibco)	10%	-	37°C, 5% CO_2	1/12-1/15
SKUT1	HTB-114	Leiomyosarcoma	eagles minimum essential (Glibco)	10%	-	37°C, 5% CO_2	1/10-1/12
sNF96.2	CRL-2884	MPNST (NF1)	dulbecco's modified eagles (Glibco)	10%	-	37°C, 5% CO_2	1/2
93T449	CRL-3043	Well differentiated liposarcoma	RPMI-1640 (Glibco)	10%	-	37°C, 5% CO_2	1/3-1/4
SW684	HTB-91	Fibrosarcoma	leibovitz's L-15 (Glibco)	10%	2%	37°C, 5% CO_2	1/2-1/3
SW872	HTB-92	Liposarcoma	leibovitz's L-15 (Glibco)	10%	2%	37°C, 5% CO_2	1/6-1/8
SW982	HTB-93	Synovial sarcoma	leibovitz's L-15 (Glibco)	10%	2%	37°C, 5% CO_2	1/3-1/4

Supplementary Table 2: Genes induced by hypoxia in all seven soft tissue sarcoma cell lines

ENSMBL	Gene name	Type
ENSG00000059804.15	SLC2A3	protein_coding
ENSG00000072682.18	P4HA2	protein_coding
ENSG00000088340.15	FER1L4	unitary_pseudogene
ENSG00000100027.14	YPEL1	protein_coding
ENSG00000104419.14	NDRG1	protein_coding
ENSG00000104765.14	BNIP3L	protein_coding
ENSG00000109107.13	ALDOC	protein_coding
ENSG00000111674.8	ENO2	protein_coding
ENSG00000112379.8	ARFGEF3	protein_coding
ENSG00000112715.20	VEGFA	protein_coding
ENSG00000114023.15	FAM162A	protein_coding
ENSG00000114268.11	PFKFB4	protein_coding
ENSG00000114480.12	GBE1	protein_coding
ENSG00000117266.15	CDK18	protein_coding
ENSG00000117394.19	SLC2A1	protein_coding
ENSG00000119950.20	MXI1	protein_coding
ENSG00000122884.12	P4HA1	protein_coding
ENSG00000123384.13	LRP1	protein_coding
ENSG00000125629.14	INSIG2	protein_coding
ENSG00000134107.4	BHLHE40	protein_coding
ENSG00000143590.13	EFNA3	protein_coding
ENSG00000143847.15	PPFIA4	protein_coding
ENSG00000147852.15	VLDLR	protein_coding
ENSG00000151006.7	PRSS53	protein_coding
ENSG00000152256.13	PDK1	protein_coding
ENSG00000159399.9	HK2	protein_coding
ENSG00000163516.13	ANKZF1	protein_coding
ENSG00000164849.8	GPR146	protein_coding
ENSG00000165507.8	C10orf10	protein_coding
ENSG00000168209.4	DDIT4	protein_coding
ENSG00000176171.11	BNIP3	protein_coding
ENSG00000186352.8	ANKRD37	protein_coding
ENSG00000186918.13	ZNF395	protein_coding
ENSG00000196968.10	FUT11	protein_coding
ENSG00000214274.9	ANG	protein_coding
ENSG00000228288.6	PCAT6	antisense
ENSG00000272870.1	RP11-798M19.6	antisense

Supplementary Table 3: Gene ontology terms enriched with protein coding genes induced by hypoxia in more than five cell lines

Ontology term	Nominal *P*	Fold enrichment	Benjamini
Response to hypoxia	1.13E-11	11.24	9.59E-09
Canonical glycolysis	2.34E-11	41.82	9.90E-09
Glycolytic process	9.71E-09	28.42	2.74E-06
Cellular response to hypoxia	1.22E-06	11.33	2.59E-04
Gluconeogenesis	2.93E-05	16.47	0.004944
Fructose metabolic process	6.25E-05	48.32	0.008781
Cellular protein modification process	2.28E-04	8.05	0.027246
Peptidyl-proline hydroxylation to 4-hydroxy-L-proline	6.60E-04	72.48	0.067487
Regulation of glucose metabolic process	7.46E-04	21.96	0.067814
Positive regulation of apoptotic process	8.43E-04	4.027	0.068955
Extracellular matrix organization	0.0011863	4.93	0.087349
Oxidation-reduction process	0.0011958	2.86	0.080984
Glycogen biosynthetic process	0.0012294	18.59	0.077023
Circadian regulation of gene expression	0.001231	10.60	0.07181
Regulation of transcription from RNA Polymerase II promoter in response to hypoxia	0.0015306	17.26	0.082859
Glycogen metabolic process	0.001697	16.66	0.085988

Benjamini: multiple test corrected P values using Benjamini method.

Supplementary Table 4: KEGG pathways enriched with protein coding genes induced by hypoxia in more than five cell lines

KEGG	*P*	Fold enrichment	Benjamini
HIF-1 signaling pathway	2.66E-07	10.68336	2.36E-05
Glycolysis / Gluconeogenesis	2.61E-06	12.50113	1.16E-04
Fructose and mannose metabolism	1.04E-05	19.63068	3.09E-04
Biosynthesis of antibiotics	1.46E-04	4.938536	0.003244
Circadian rhythm	1.85E-04	16.88661	0.003295
Carbon metabolism	6.31E-04	6.485653	0.009317
Starch and sucrose metabolism	0.003544	12.69054	0.044136
Biosynthesis of amino acids	0.005013	7.07412	0.054371
Axon guidance	0.006637	4.946314	0.063726

Benjamini: multiple test corrected *P* values using Benjamini method.

Supplementary Table 5: Gene ontology and KEGG pathways enriched with genes down-regulated by hypoxia in more than four cell lines

Term	*P*	Fold Enrichment	Benjamini
nucleolus	4.68E-15	4.7975638	8.72E-13
nucleoplasm	9.71E-13	2.5146131	9.08E-11
DNA replication initiation	2.84E-13	35.412577	2.45E-10
DNA replication	3.07E-10	9.9695231	1.33E-07
G1/S transition of mitotic cell cycle	4.01E-09	12.119812	1.15E-06
rRNA processing	2.04E-08	7.2209162	4.41E-06
preribosome, large subunit precursor	5.37E-06	22.990749	3.35E-04
poly(A) RNA binding	1.89E-06	2.7271254	5.59E-04
Cell cycle	5.97E-06	7.4301075	8.35E-04
replication fork protection complex	2.39E-05	63.498258	0.001119
DNA replication	3.58E-05	15.355556	0.0025
small-subunit processome	2.60E-04	15.874564	0.009684
Ribosome biogenesis in eukaryotes	3.13E-04	7.4130268	0.014482
Pyrimidine metabolism	8.14E-04	6.2012821	0.028094
regulation of cell cycle	2.00E-04	6.6463487	0.033918
nucleus	0.001219	1.3954372	0.037302
mitochondrion	0.001754	2.0037016	0.045819
mitochondrial inner membrane	0.002098	3.0237266	0.047904

Benjamini: multiple test corrected P values using Benjamini method.

Supplementary Table 6: Pathways identified by GSEA as enriched in high-hypoxia tumors from the French training cohort. See Supplementary_Table_6

Supplementary Table 7: The 24-gene hypoxia signature derived in this work

Gene name
ENO2
SLC2A1
BNIP3
PDK1
NDRG1
PFKFB4
FAM162A
VEGFA
ZNF395
DDIT4
ANKRD37
MXI1
SLC2A3
PPFIA4
GBE1
ALDOC
CDK18
ANG
PRSS53
INSIG2
VLDLR
P4HA1
BNIP3L
BHLHE40

Supplementary Table 8: Assignment of de novo hypoxia signature. Supplementary_Table_8

Supplementary Table 9: Association between the *de novo* hypoxia signature and clinic-pathological factors

		**French**		
		**Low-hypoxia**	**High-hypoxia**	***P***
diagnosis	Liposarcoma	53	9	0.03
	Leiomyosarcoma	60	24	
	Other	17	10	
	Undifferentiated sarcoma	111	25	
tissue site	Extremities	147	40	0.2
	Head and neck	3	0	
	Internal trunk	54	11	
	Trunk wall	37	17	
		TCGA		
		Low-hypoxia	High-hypoxia	P
diagnosis	liposarcoma	54	4	0.004
	Desmoid tumor	2	0	
	Leiomyosarcoma	73	31	
	Malignant Peripheral Nerve Sheath Tumors (MPNST)	6	3	
	Myxofibrosarcoma	23	2	
	Pleomorphic MFH/ Undifferentiated pleomorphic sarcoma	40	10	
	Synovial Sarcoma	10	0	
tissue site	Extremity	64	21	0.72
	Abdomen	21	5	
	Breast	1	0	
	Head and Neck	4	1	
	Pelvic	11	3	
	Retroperitoneal	62	9	
	Thorax	18	6	
	Uterine	26	5	
gender	FEMALE	113	28	0.96
	MALE	95	22	
age		20–90 (61)	20–88 (60.5)	0.8
pathologic length lesion		1.2–121 (10.9)	3.5–32 (9.2)	0.15

Supplementary Table 10: Association between hypoxia and CINSARC signatures

	**Low-hypoxia**	**High-hypoxia**
Low CINSARC	73	10
High CINSARC	68	31

Supplementary Table 11: GO terms and pathways identified by GSEA as enriched with genes down-regulated in high-hypoxia tumors. See Supplementary_Table_11

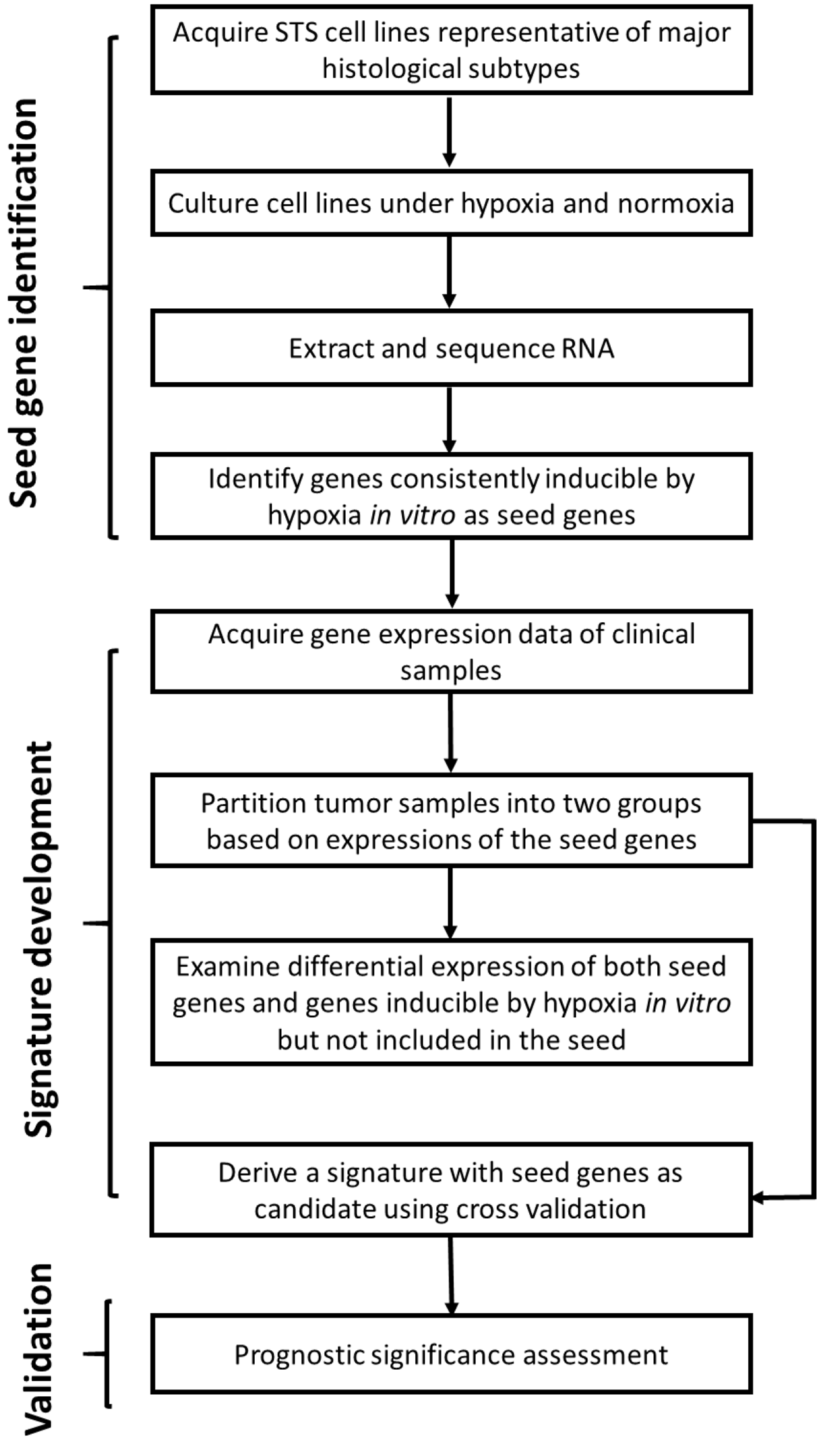

Supplementary Figure 1: Schematic presentation of the study design.

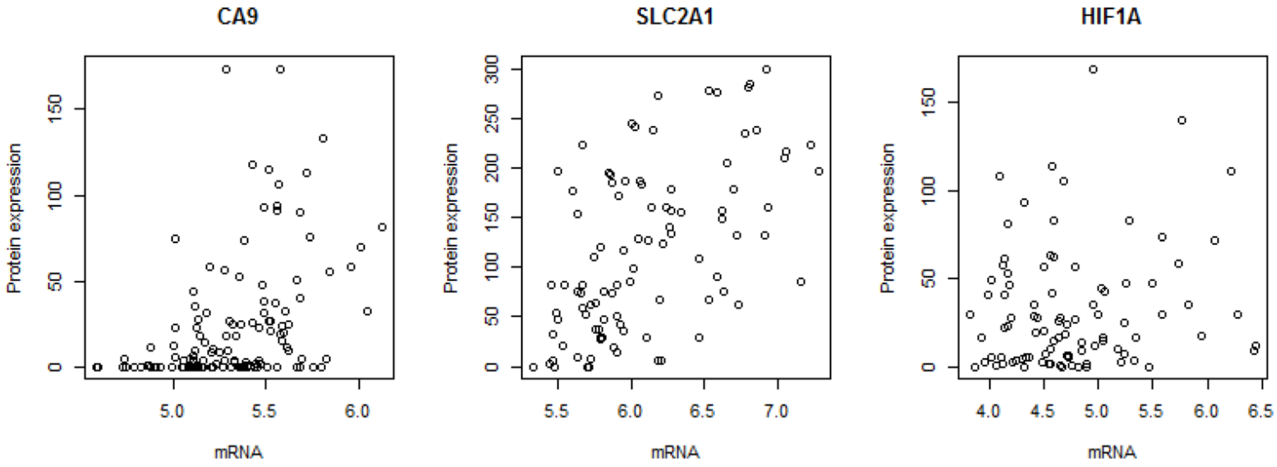

Supplementary Figure 2: In a cohort of urothelial cancer patients, mRNA level and protein expression are strongly correlated for *CA9* and *SLC2A1*, but not *HIF1A*.

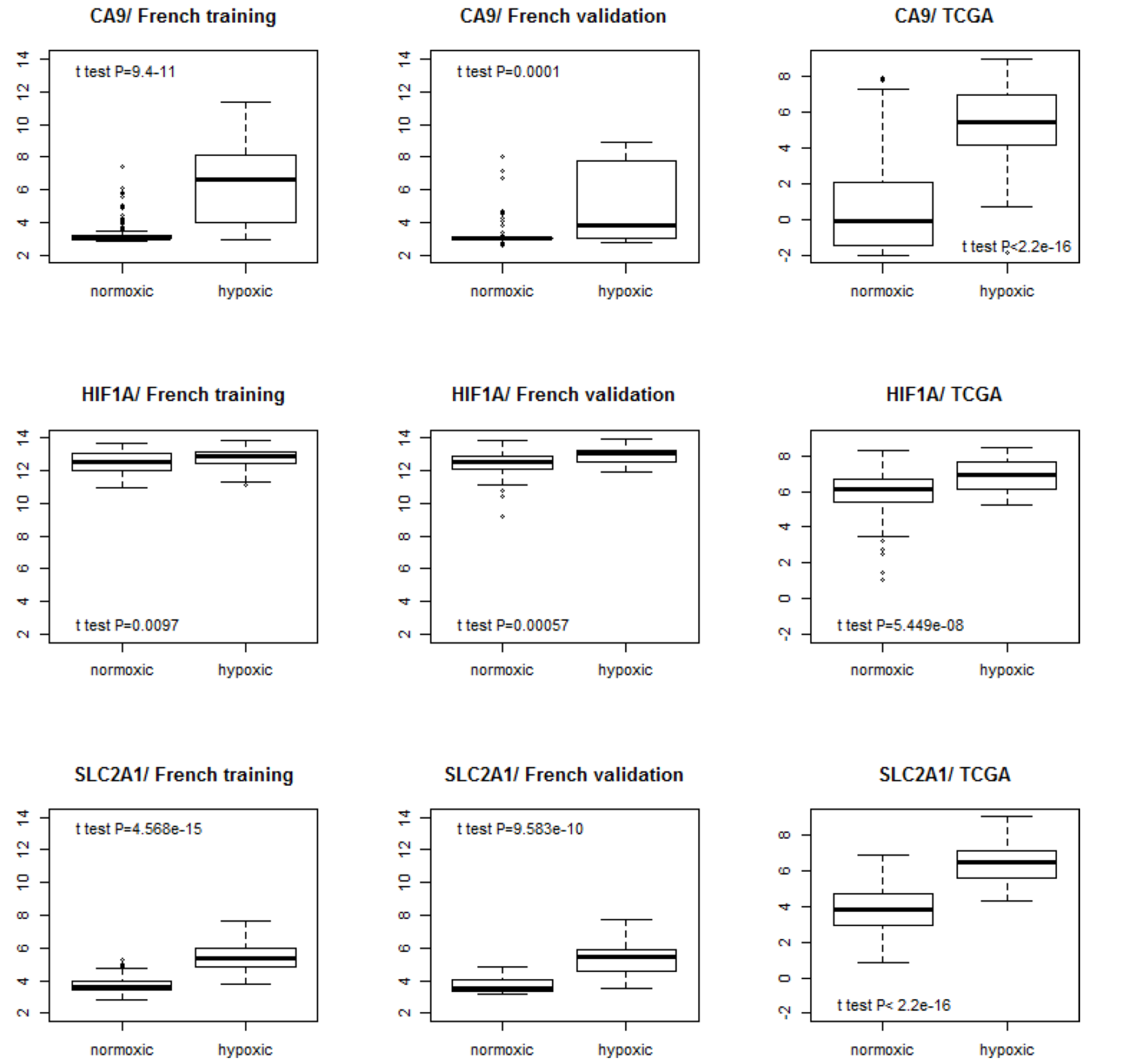

Supplementary Figure 3: Association between the de novo 24-gene signature and mRNA expression of *CA9, HIF1A* and *SLC2A1*.

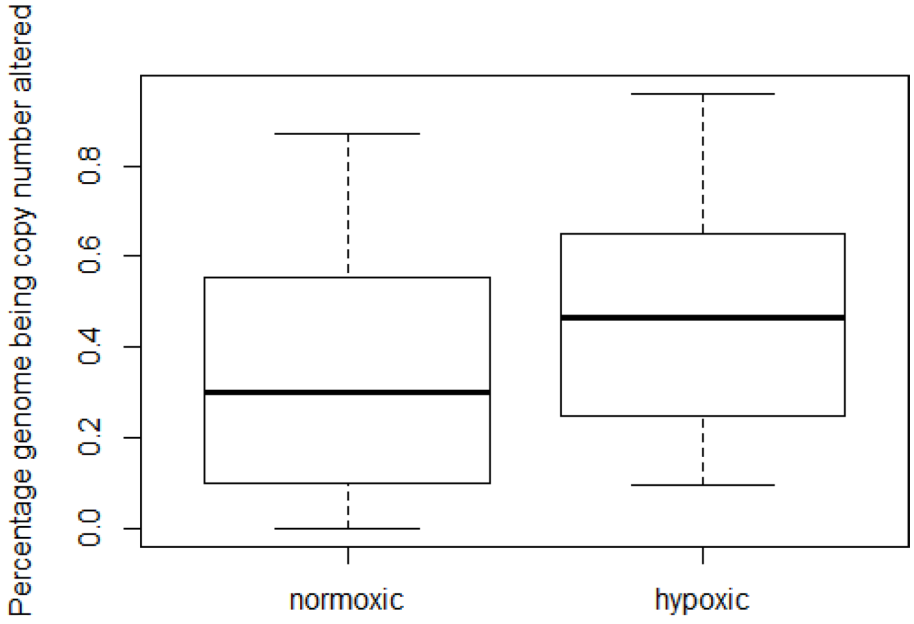

Supplementary Figure 4: Association between global copy number alteration burden and the *de novo* hypoxia signature in the TCGA cohort.

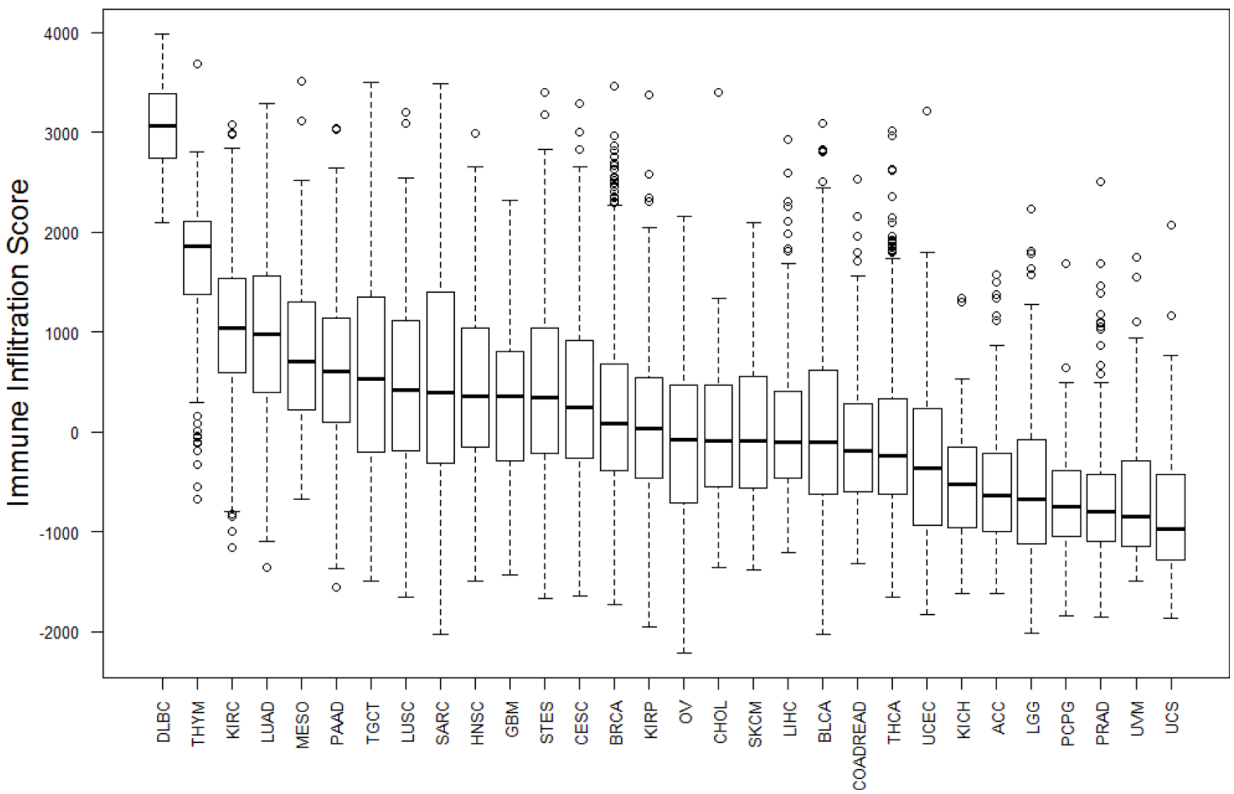

Supplementary Figure 5: Pan-cancer analysis of immune cell infiltration scores produced by ESTIMATE.

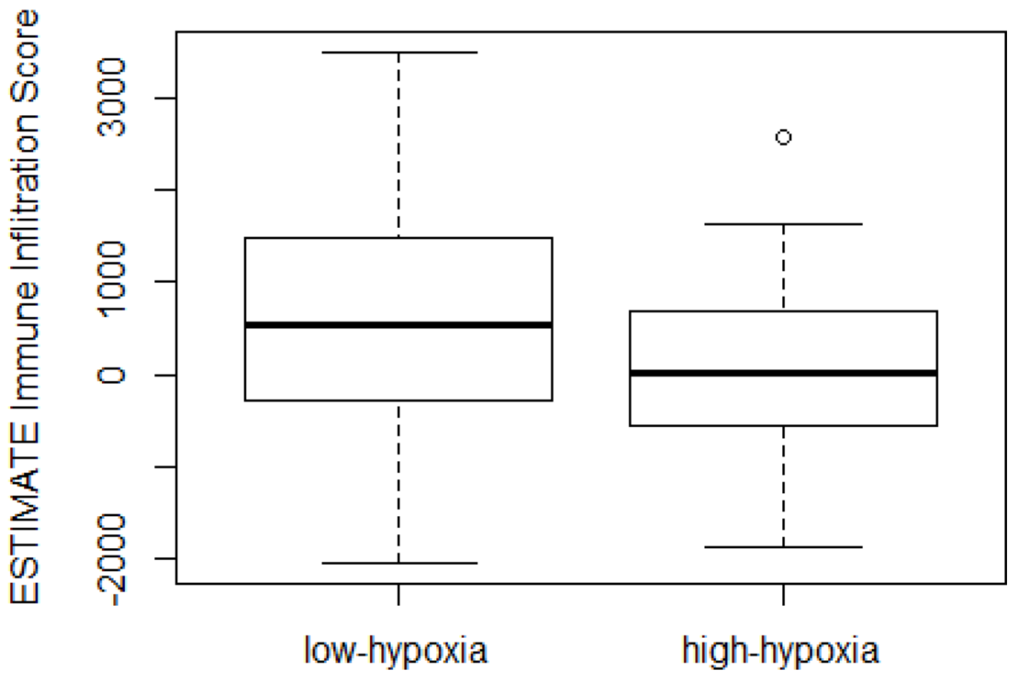

Supplementary Figure 6: Distribution of immune infiltration scores predicted by ESTIMATE across high-hypoxia and low-hypoxia STS tumors.

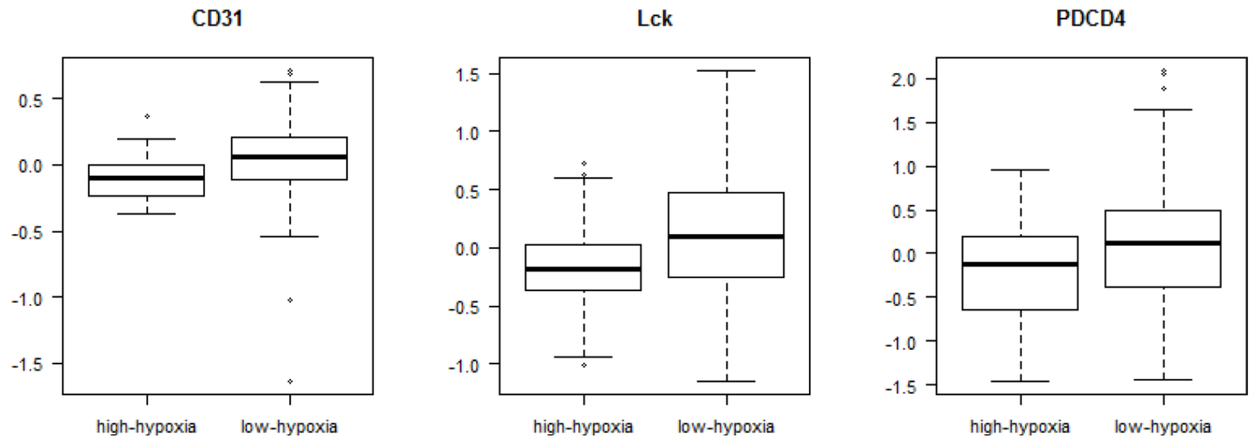

Supplementary Figure 7: Distribution of three immune related protein expression across high-hypoxia and low-hypoxia signature in TCGA cohort.
