# Supplementary material for: Validation of a hypoxia related gene signature in multiple soft tissue sarcoma cohorts
Source: Oncotarget. 2017 Dec 12;9(3):3946–55. doi: 10.18632/oncotarget.23280 (PMC5790513; doi:10.18632/oncotarget.23280)
Supplement: Supplementary file 2 [file oncotarget-09-3946-s002.docx]

**Supplementary Table S6.** Pathways identified by GSEA as enriched in high-hypoxia tumors from the French training cohort

| NAME | SIZE | NES | FDR q-val |
| --- | --- | --- | --- |
| ROSTY_CERVICAL_CANCER_PROLIFERATION_CLUSTER | 136 | 2.772621 | 0 |
| MENSE_HYPOXIA_UP | 94 | 2.729226 | 0 |
| SOTIRIOU_BREAST_CANCER_GRADE_1_VS_3_UP | 140 | 2.593809 | 0 |
| HARRIS_HYPOXIA | 78 | 2.59126 | 0 |
| ELVIDGE_HYPOXIA_BY_DMOG_UP | 123 | 2.589598 | 0 |
| SHEDDEN_LUNG_CANCER_POOR_SURVIVAL_A6 | 424 | 2.583794 | 0 |
| ELVIDGE_HIF1A_TARGETS_DN | 86 | 2.582839 | 0 |
| NAKAYAMA_SOFT_TISSUE_TUMORS_PCA2_UP | 86 | 2.581241 | 0 |
| WINTER_HYPOXIA_UP | 81 | 2.578937 | 0 |
| WINTER_HYPOXIA_METAGENE | 226 | 2.557733 | 0 |
| KOBAYASHI_EGFR_SIGNALING_24HR_DN | 234 | 2.546267 | 0 |
| ELVIDGE_HIF1A_AND_HIF2A_TARGETS_DN | 99 | 2.546146 | 0 |
| ELVIDGE_HYPOXIA_UP | 159 | 2.529292 | 0 |
| QI_HYPOXIA | 133 | 2.527242 | 0 |
| VANTVEER_BREAST_CANCER_METASTASIS_DN | 108 | 2.524836 | 0 |
| CHANG_CYCLING_GENES | 137 | 2.509404 | 0 |
| KIM_HYPOXIA | 24 | 2.448844 | 0 |
| SEMENZA_HIF1_TARGETS | 34 | 2.439188 | 0 |
| LEONARD_HYPOXIA | 45 | 2.409583 | 0 |
| VECCHI_GASTRIC_CANCER_EARLY_UP | 384 | 2.39393 | 0 |
| ZHAN_MULTIPLE_MYELOMA_PR_UP | 43 | 2.387739 | 0 |
| FARDIN_HYPOXIA_11 | 30 | 2.377227 | 0 |
| LEE_EARLY_T_LYMPHOCYTE_UP | 96 | 2.367318 | 0 |
| CROONQUIST_NRAS_SIGNALING_DN | 72 | 2.36024 | 0 |
| KONG_E2F3_TARGETS | 90 | 2.359015 | 0 |
| REACTOME_GLYCOLYSIS | 26 | 2.341999 | 0 |
| KANG_DOXORUBICIN_RESISTANCE_UP | 52 | 2.341144 | 0 |
| DUTERTRE_ESTRADIOL_RESPONSE_24HR_UP | 302 | 2.328301 | 0 |
| REACTOME_GLUCOSE_METABOLISM | 62 | 2.324387 | 0 |
| WINNEPENNINCKX_MELANOMA_METASTASIS_UP | 151 | 2.31927 | 0 |
| SHIPP_DLBCL_VS_FOLLICULAR_LYMPHOMA_UP | 42 | 2.297002 | 0 |
| WHITEFORD_PEDIATRIC_CANCER_MARKERS | 110 | 2.295661 | 0 |
| MISSIAGLIA_REGULATED_BY_METHYLATION_DN | 114 | 2.283461 | 0 |
| RUIZ_TNC_TARGETS_DN | 131 | 2.271131 | 0 |
| PRAMOONJAGO_SOX4_TARGETS_UP | 51 | 2.270425 | 0 |
| MORI_IMMATURE_B_LYMPHOCYTE_DN | 87 | 2.263043 | 0 |
| CHEMNITZ_RESPONSE_TO_PROSTAGLANDIN_E2_UP | 131 | 2.260914 | 0 |
| ODONNELL_TFRC_TARGETS_DN | 129 | 2.250758 | 0 |
| FOURNIER_ACINAR_DEVELOPMENT_LATE_2 | 256 | 2.234073 | 0 |
| KEGG_FRUCTOSE_AND_MANNOSE_METABOLISM | 33 | 2.231426 | 0 |
| RHODES_UNDIFFERENTIATED_CANCER | 66 | 2.225542 | 0 |
| CROONQUIST_IL6_DEPRIVATION_DN | 98 | 2.22504 | 0 |
| CHIANG_LIVER_CANCER_SUBCLASS_PROLIFERATION_UP | 164 | 2.22369 | 0 |
| MARTORIATI_MDM4_TARGETS_NEUROEPITHELIUM_UP | 155 | 2.223373 | 0 |
| HOFFMANN_LARGE_TO_SMALL_PRE_BII_LYMPHOCYTE_UP | 149 | 2.222841 | 0 |
| PID_HIF1_TFPATHWAY | 65 | 2.212369 | 0 |
| FERREIRA_EWINGS_SARCOMA_UNSTABLE_VS_STABLE_UP | 149 | 2.199161 | 0 |
| WANG_RESPONSE_TO_GSK3_INHIBITOR_SB216763_DN | 323 | 2.197625 | 0 |
| SARRIO_EPITHELIAL_MESENCHYMAL_TRANSITION_UP | 161 | 2.190648 | 0 |
| MARTORIATI_MDM4_TARGETS_FETAL_LIVER_UP | 201 | 2.18686 | 0 |
| WU_APOPTOSIS_BY_CDKN1A_VIA_TP53 | 52 | 2.182998 | 0 |
| BURTON_ADIPOGENESIS_3 | 101 | 2.179957 | 0 |
| WONG_EMBRYONIC_STEM_CELL_CORE | 318 | 2.17703 | 0 |
| WHITFIELD_CELL_CYCLE_LITERATURE | 44 | 2.171037 | 0 |
| MOOTHA_GLYCOLYSIS | 20 | 2.155306 | 0 |
| BASAKI_YBX1_TARGETS_UP | 267 | 2.153959 | 0 |
| GREENBAUM_E2A_TARGETS_UP | 33 | 2.145845 | 0 |
| FARMER_BREAST_CANCER_CLUSTER_2 | 32 | 2.140396 | 0 |
| VANDESLUIS_COMMD1_TARGETS_GROUP_3_UP | 84 | 2.129941 | 0 |
| AMUNDSON_GAMMA_RADIATION_RESPONSE | 39 | 2.129818 | 0 |
| ZHOU_CELL_CYCLE_GENES_IN_IR_RESPONSE_6HR | 82 | 2.126611 | 0 |
| KRIEG_HYPOXIA_VIA_KDM3A | 50 | 2.120327 | 0 |
| LIEN_BREAST_CARCINOMA_METAPLASTIC_VS_DUCTAL_UP | 76 | 2.117474 | 0 |
| WANG_ADIPOGENIC_GENES_REPRESSED_BY_SIRT1 | 25 | 2.116553 | 0 |
| FURUKAWA_DUSP6_TARGETS_PCI35_DN | 66 | 2.115399 | 0 |
| ODONNELL_TARGETS_OF_MYC_AND_TFRC_DN | 45 | 2.111408 | 0 |
| ZHANG_TLX_TARGETS_DN | 83 | 2.093721 | 3.12E-05 |
| FRASOR_RESPONSE_TO_SERM_OR_FULVESTRANT_DN | 49 | 2.089645 | 3.08E-05 |
| NIELSEN_LEIOMYOSARCOMA_CNN1_UP | 19 | 2.087955 | 3.03E-05 |
| MOLENAAR_TARGETS_OF_CCND1_AND_CDK4_DN | 51 | 2.086041 | 2.99E-05 |
| REACTOME_MUSCLE_CONTRACTION | 45 | 2.083972 | 4.43E-05 |
| REACTOME_SMOOTH_MUSCLE_CONTRACTION | 22 | 2.083461 | 4.37E-05 |
| REACTOME_GLUCONEOGENESIS | 31 | 2.081169 | 5.71E-05 |
| MORI_LARGE_PRE_BII_LYMPHOCYTE_UP | 82 | 2.073474 | 7.02E-05 |
| KEGG_PENTOSE_PHOSPHATE_PATHWAY | 26 | 2.060951 | 1.11E-04 |
| DELPUECH_FOXO3_TARGETS_DN | 39 | 2.060713 | 1.09E-04 |
| REICHERT_MITOSIS_LIN9_TARGETS | 26 | 2.059284 | 1.21E-04 |
| GRAHAM_CML_DIVIDING_VS_NORMAL_QUIESCENT_UP | 177 | 2.05798 | 1.20E-04 |
| ZHANG_TLX_TARGETS_60HR_DN | 253 | 2.056499 | 1.32E-04 |
| GRAHAM_NORMAL_QUIESCENT_VS_NORMAL_DIVIDING_DN | 86 | 2.054364 | 1.43E-04 |
| GAVIN_FOXP3_TARGETS_CLUSTER_P6 | 87 | 2.054354 | 1.41E-04 |
| ISHIDA_E2F_TARGETS | 51 | 2.054121 | 1.39E-04 |
| MOOTHA_GLUCONEOGENESIS | 32 | 2.051652 | 1.50E-04 |
| FOURNIER_ACINAR_DEVELOPMENT_LATE_DN | 20 | 2.042302 | 1.61E-04 |
| OXFORD_RALA_OR_RALB_TARGETS_UP | 45 | 2.014794 | 2.69E-04 |
| BENPORATH_PROLIFERATION | 136 | 2.005694 | 3.51E-04 |
| HORIUCHI_WTAP_TARGETS_DN | 288 | 2.004785 | 3.59E-04 |
| NIELSEN_LIPOSARCOMA_DN | 19 | 2.001899 | 3.55E-04 |
| PID_PLK1_PATHWAY | 43 | 1.992884 | 3.86E-04 |
| SONG_TARGETS_OF_IE86_CMV_PROTEIN | 59 | 1.992636 | 3.81E-04 |
| YU_MYC_TARGETS_UP | 39 | 1.990344 | 3.88E-04 |
| GROSS_HIF1A_TARGETS_DN | 22 | 1.989183 | 3.96E-04 |
| KEGG_GLYCOLYSIS_GLUCONEOGENESIS | 60 | 1.988793 | 3.92E-04 |
| MANALO_HYPOXIA_UP | 199 | 1.975619 | 5.65E-04 |
| KAMMINGA_EZH2_TARGETS | 41 | 1.970872 | 6.14E-04 |
| KAUFFMANN_MELANOMA_RELAPSE_UP | 56 | 1.964084 | 6.62E-04 |
| BOYAULT_LIVER_CANCER_SUBCLASS_G3_UP | 177 | 1.960707 | 7.09E-04 |
| TANG_SENESCENCE_TP53_TARGETS_DN | 57 | 1.954325 | 8.39E-04 |
| CHANG_CORE_SERUM_RESPONSE_UP | 199 | 1.949775 | 9.04E-04 |
| KEGG_STARCH_AND_SUCROSE_METABOLISM | 34 | 1.94775 | 9.16E-04 |
| REACTOME_REGULATION_OF_HYPOXIA_INDUCIBLE_FACTOR_HIF_BY_OXYGEN | 23 | 1.943826 | 0.001041 |
| REACTOME_CHROMOSOME_MAINTENANCE | 98 | 1.942287 | 0.001041 |
| KAN_RESPONSE_TO_ARSENIC_TRIOXIDE | 119 | 1.935722 | 0.001173 |
| EGUCHI_CELL_CYCLE_RB1_TARGETS | 23 | 1.934484 | 0.001212 |
| SMIRNOV_RESPONSE_TO_IR_6HR_DN | 109 | 1.932369 | 0.00126 |
| SABATES_COLORECTAL_ADENOMA_UP | 126 | 1.93184 | 0.001258 |
| BLUM_RESPONSE_TO_SALIRASIB_DN | 324 | 1.931785 | 0.001246 |
| MITSIADES_RESPONSE_TO_APLIDIN_DN | 236 | 1.931128 | 0.001283 |
| GROSS_HYPOXIA_VIA_ELK3_AND_HIF1A_UP | 137 | 1.928592 | 0.001319 |
| LANDIS_ERBB2_BREAST_PRENEOPLASTIC_UP | 18 | 1.923963 | 0.001449 |
| OXFORD_RALA_OR_RALB_TARGETS_DN | 21 | 1.921057 | 0.001549 |
| OLSSON_E2F3_TARGETS_DN | 43 | 1.920617 | 0.001573 |
| GRAESSMANN_RESPONSE_TO_MC_AND_SERUM_DEPRIVATION_DN | 75 | 1.91999 | 0.001596 |
| HOOI_ST7_TARGETS_DN | 110 | 1.913579 | 0.00191 |
| RICKMAN_TUMOR_DIFFERENTIATED_WELL_VS_MODERATELY_DN | 102 | 1.904257 | 0.002168 |
| WEST_ADRENOCORTICAL_TUMOR_MARKERS_UP | 21 | 1.901338 | 0.002212 |
| PUJANA_BRCA_CENTERED_NETWORK | 114 | 1.900447 | 0.002256 |
| GRAESSMANN_APOPTOSIS_BY_SERUM_DEPRIVATION_DN | 213 | 1.898702 | 0.002298 |
| CREIGHTON_AKT1_SIGNALING_VIA_MTOR_DN | 22 | 1.897483 | 0.002331 |
| REACTOME_DEPOSITION_OF_NEW_CENPA_CONTAINING_NUCLEOSOMES_AT_THE_CENTROMERE | 43 | 1.897245 | 0.002329 |
| SMID_BREAST_CANCER_LUMINAL_A_DN | 17 | 1.896513 | 0.002336 |
| PUJANA_XPRSS_INT_NETWORK | 157 | 1.879695 | 0.003361 |
| SHAFFER_IRF4_TARGETS_IN_ACTIVATED_B_LYMPHOCYTE | 68 | 1.879217 | 0.003376 |
| REN_BOUND_BY_E2F | 59 | 1.879194 | 0.003349 |
| PEDERSEN_TARGETS_OF_611CTF_ISOFORM_OF_ERBB2 | 70 | 1.877679 | 0.00343 |
| LE_EGR2_TARGETS_UP | 105 | 1.874444 | 0.003576 |
| LY_AGING_MIDDLE_DN | 16 | 1.871823 | 0.003712 |
| PID_HIF2PATHWAY | 32 | 1.869956 | 0.003814 |
| SCIAN_CELL_CYCLE_TARGETS_OF_TP53_AND_TP73_DN | 22 | 1.865569 | 0.004091 |
| MIYAGAWA_TARGETS_OF_EWSR1_ETS_FUSIONS_DN | 201 | 1.865228 | 0.004116 |
| ZAMORA_NOS2_TARGETS_UP | 60 | 1.855308 | 0.004825 |
| FINETTI_BREAST_CANCER_KINOME_RED | 16 | 1.847636 | 0.005271 |
| KEGG_DNA_REPLICATION | 36 | 1.843635 | 0.00564 |
| NAKAMURA_CANCER_MICROENVIRONMENT_DN | 42 | 1.842256 | 0.005737 |
| LANDIS_BREAST_CANCER_PROGRESSION_UP | 43 | 1.840163 | 0.005826 |
| BENPORATH_ES_1 | 346 | 1.839361 | 0.005844 |
| VERNELL_RETINOBLASTOMA_PATHWAY_UP | 67 | 1.838275 | 0.005947 |
| LI_WILMS_TUMOR_VS_FETAL_KIDNEY_1_DN | 152 | 1.837221 | 0.005978 |
| QI_HYPOXIA_TARGETS_OF_HIF1A_AND_FOXA2 | 34 | 1.835512 | 0.006107 |
| YOKOE_CANCER_TESTIS_ANTIGENS | 31 | 1.833583 | 0.006256 |
| MANALO_HYPOXIA_DN | 264 | 1.826833 | 0.006901 |
| MORI_PRE_BI_LYMPHOCYTE_UP | 76 | 1.825082 | 0.007028 |
| PID_AURORA_B_PATHWAY | 39 | 1.824365 | 0.007052 |
| SIMBULAN_PARP1_TARGETS_DN | 17 | 1.823556 | 0.007111 |
| HENDRICKS_SMARCA4_TARGETS_UP | 52 | 1.822728 | 0.007134 |
| LINDGREN_BLADDER_CANCER_CLUSTER_3_UP | 298 | 1.822521 | 0.00712 |
| DANG_REGULATED_BY_MYC_UP | 64 | 1.821507 | 0.007179 |
| GARGALOVIC_RESPONSE_TO_OXIDIZED_PHOSPHOLIPIDS_RED_UP | 17 | 1.819385 | 0.007382 |
| MATTHEWS_AP1_TARGETS | 16 | 1.819157 | 0.007375 |
| BOYAULT_LIVER_CANCER_SUBCLASS_G23_UP | 48 | 1.818352 | 0.007402 |
| BIDUS_METASTASIS_UP | 202 | 1.816538 | 0.007525 |
| GHANDHI_BYSTANDER_IRRADIATION_UP | 77 | 1.815944 | 0.007578 |
| BROWNE_HCMV_INFECTION_2HR_DN | 48 | 1.81532 | 0.007611 |
| WELCSH_BRCA1_TARGETS_DN | 133 | 1.814874 | 0.007595 |
| REACTOME_TELOMERE_MAINTENANCE | 59 | 1.813176 | 0.007727 |
| KIM_WT1_TARGETS_8HR_UP | 158 | 1.809537 | 0.008184 |
| ZHOU_CELL_CYCLE_GENES_IN_IR_RESPONSE_24HR | 121 | 1.809076 | 0.008204 |
| FUJII_YBX1_TARGETS_DN | 195 | 1.80602 | 0.008454 |
| SU_TESTIS | 74 | 1.805539 | 0.00844 |
| JEON_SMAD6_TARGETS_DN | 18 | 1.804783 | 0.008544 |
| SCIBETTA_KDM5B_TARGETS_DN | 76 | 1.802617 | 0.008847 |
| RHODES_CANCER_META_SIGNATURE | 63 | 1.802032 | 0.008876 |
| SUNG_METASTASIS_STROMA_DN | 47 | 1.800779 | 0.008988 |
| MOOTHA_PGC | 395 | 1.799984 | 0.009016 |
| LIAO_METASTASIS | 495 | 1.799278 | 0.009075 |
| AMIT_EGF_RESPONSE_480_MCF10A | 41 | 1.796628 | 0.00936 |
| GRADE_COLON_AND_RECTAL_CANCER_UP | 262 | 1.796569 | 0.009311 |
| CHIARADONNA_NEOPLASTIC_TRANSFORMATION_KRAS_CDC25_UP | 55 | 1.792634 | 0.009665 |
| NES: normalized enrichment score; FDR q-val: false discovery rate corrected P value | | | |
