# Supplementary material for: Validation of a hypoxia related gene signature in multiple soft tissue sarcoma cohorts
Source: Oncotarget. 2017 Dec 12;9(3):3946–55. doi: 10.18632/oncotarget.23280 (PMC5790513; doi:10.18632/oncotarget.23280)
Supplement: Supplementary file 3 [file oncotarget-09-3946-s003.docx]

**Supplementary Table S8.** Assignment of *de novo* hypoxia signature

|  | Hypoxia signature |  | Hypoxia signature |
| --- | --- | --- | --- |
| GSM525806 | normoxic | TCGA.DX.A1L4 | normoxic |
| GSM525807 | hypoxic | TCGA.DX.A8BH | normoxic |
| GSM525808 | hypoxic | TCGA.DX.A3LW | normoxic |
| GSM525809 | hypoxic | TCGA.PC.A5DM | hypoxic |
| GSM525810 | normoxic | TCGA.IS.A3KA | normoxic |
| GSM525811 | normoxic | TCGA.HB.A3L4 | normoxic |
| GSM525812 | hypoxic | TCGA.DX.AB3B | normoxic |
| GSM525813 | normoxic | TCGA.X6.A7WC | hypoxic |
| GSM525814 | normoxic | TCGA.3B.A9HV | hypoxic |
| GSM525815 | normoxic | TCGA.3B.A9HY | normoxic |
| GSM525816 | normoxic | TCGA.Z4.A9VC | normoxic |
| GSM525817 | normoxic | TCGA.WK.A8XY | hypoxic |
| GSM525818 | normoxic | TCGA.SI.A71O | hypoxic |
| GSM525819 | normoxic | TCGA.3B.A9HR | hypoxic |
| GSM525820 | normoxic | TCGA.DX.AB2T | normoxic |
| GSM525821 | normoxic | TCGA.X6.A8C4 | hypoxic |
| GSM525822 | normoxic | TCGA.DX.A48N | normoxic |
| GSM525823 | hypoxic | TCGA.X6.A8C5 | normoxic |
| GSM525824 | normoxic | TCGA.3B.A9HS | normoxic |
| GSM525825 | normoxic | TCGA.DX.AB30 | normoxic |
| GSM525826 | normoxic | TCGA.IW.A3M6 | normoxic |
| GSM525827 | hypoxic | TCGA.3B.A9HQ | normoxic |
| GSM525828 | normoxic | TCGA.DX.A6BG | normoxic |
| GSM525829 | hypoxic | TCGA.DX.A48R | normoxic |
| GSM525830 | normoxic | TCGA.X6.A7WA | normoxic |
| GSM525831 | normoxic | TCGA.PT.A8TR | normoxic |
| GSM525832 | hypoxic | TCGA.DX.AB2Z | normoxic |
| GSM525833 | hypoxic | TCGA.DX.A3UD | hypoxic |
| GSM525834 | normoxic | TCGA.RN.A68Q | normoxic |
| GSM525835 | normoxic | TCGA.PC.A5DP | hypoxic |
| GSM525836 | normoxic | TCGA.DX.A7EO | normoxic |
| GSM525837 | normoxic | TCGA.DX.A8BK | normoxic |
| GSM525838 | hypoxic | TCGA.DX.AB2P | normoxic |
| GSM525839 | hypoxic | TCGA.DX.A6BB | normoxic |
| GSM525840 | normoxic | TCGA.DX.AB2O | normoxic |
| GSM525841 | hypoxic | TCGA.X6.A7W8 | normoxic |
| GSM525842 | hypoxic | TCGA.HS.A5N8 | normoxic |
| GSM525843 | hypoxic | TCGA.K1.A6RU | normoxic |
| GSM525844 | normoxic | TCGA.K1.A3PN | normoxic |
| GSM525845 | normoxic | TCGA.DX.A8BJ | hypoxic |
| GSM525846 | hypoxic | TCGA.K1.A42X | normoxic |
| GSM525847 | hypoxic | TCGA.DX.A6B8 | hypoxic |
| GSM525848 | normoxic | TCGA.MJ.A850 | normoxic |
| GSM525849 | normoxic | TCGA.QQ.A8VD | normoxic |
| GSM525850 | normoxic | TCGA.Z4.AAPG | normoxic |
| GSM525851 | hypoxic | TCGA.DX.A8BU | hypoxic |
| GSM525852 | hypoxic | TCGA.DX.A1L2 | normoxic |
| GSM525853 | hypoxic | TCGA.MJ.A68J | normoxic |
| GSM525854 | normoxic | TCGA.VT.AB3D | normoxic |
| GSM525855 | normoxic | TCGA.DX.A3UC | normoxic |
| GSM525856 | normoxic | TCGA.MB.A5YA | normoxic |
| GSM525857 | normoxic | TCGA.WP.A9GB | normoxic |
| GSM525858 | normoxic | TCGA.DX.A8BS | normoxic |
| GSM525859 | normoxic | TCGA.DX.A3UF | normoxic |
| GSM525860 | normoxic | TCGA.PC.A5DN | normoxic |
| GSM525861 | normoxic | TCGA.DX.A7EL | hypoxic |
| GSM525862 | normoxic | TCGA.DX.AATS | hypoxic |
| GSM525863 | normoxic | TCGA.DX.A1KY | normoxic |
| GSM525865 | normoxic | TCGA.FX.A3RE | normoxic |
| GSM525866 | normoxic | TCGA.SG.A849 | normoxic |
| GSM525867 | normoxic | TCGA.DX.A3UE | hypoxic |
| GSM525868 | hypoxic | TCGA.MB.A8JK | normoxic |
| GSM525869 | hypoxic | TCGA.DX.AB3A | normoxic |
| GSM525870 | normoxic | TCGA.DX.A6YQ | normoxic |
| GSM525871 | hypoxic | TCGA.QQ.A5V2 | normoxic |
| GSM525872 | normoxic | TCGA.DX.A48O | normoxic |
| GSM525873 | normoxic | TCGA.DX.A7EQ | normoxic |
| GSM525874 | normoxic | TCGA.DX.AB2G | normoxic |
| GSM525875 | normoxic | TCGA.KF.A41W | hypoxic |
| GSM525876 | normoxic | TCGA.QC.A6FX | hypoxic |
| GSM525877 | normoxic | TCGA.DX.A8BT | normoxic |
| GSM525878 | normoxic | TCGA.DX.AB2E | hypoxic |
| GSM525879 | hypoxic | TCGA.LI.A67I | normoxic |
| GSM525880 | normoxic | TCGA.DX.A7EM | hypoxic |
| GSM525881 | normoxic | TCGA.DX.A3U6 | normoxic |
| GSM525882 | normoxic | TCGA.SI.AA8B | hypoxic |
| GSM525883 | normoxic | TCGA.3B.A9HL | normoxic |
| GSM525884 | normoxic | TCGA.DX.A6YU | normoxic |
| GSM525885 | hypoxic | TCGA.X6.A8C3 | normoxic |
| GSM525886 | normoxic | TCGA.DX.A3LT | normoxic |
| GSM525887 | normoxic | TCGA.DX.AB35 | normoxic |
| GSM525888 | normoxic | TCGA.MO.A47R | normoxic |
| GSM525889 | normoxic | TCGA.FX.A76Y | normoxic |
| GSM525890 | normoxic | TCGA.IE.A4EK | normoxic |
| GSM525891 | normoxic | TCGA.DX.A23Y | normoxic |
| GSM525892 | normoxic | TCGA.QQ.A5V9 | normoxic |
| GSM525893 | normoxic | TCGA.3B.A9HI | hypoxic |
| GSM525894 | normoxic | TCGA.MJ.A68H | hypoxic |
| GSM525895 | normoxic | TCGA.JV.A5VF | normoxic |
| GSM525896 | normoxic | TCGA.FX.A3NJ | normoxic |
| GSM525897 | normoxic | TCGA.FX.A8OO | normoxic |
| GSM525898 | normoxic | TCGA.DX.A1L0 | normoxic |
| GSM525899 | hypoxic | TCGA.QQ.A5VA | normoxic |
| GSM525900 | normoxic | TCGA.DX.AB2F | normoxic |
| GSM525901 | normoxic | TCGA.DX.A3UA | normoxic |
| GSM525902 | normoxic | TCGA.QQ.A8VH | normoxic |
| GSM525903 | normoxic | TCGA.DX.A6BA | normoxic |
| GSM525904 | normoxic | TCGA.Z4.AAPF | normoxic |
| GSM525905 | normoxic | TCGA.X6.A7WD | hypoxic |
| GSM525906 | normoxic | TCGA.WK.A8XS | hypoxic |
| GSM525907 | normoxic | TCGA.DX.A1KW | normoxic |
| GSM525908 | normoxic | TCGA.DX.A6Z0 | normoxic |
| GSM525909 | normoxic | TCGA.DX.A1L1 | normoxic |
| GSM525910 | hypoxic | TCGA.DX.A48P | normoxic |
| GSM525911 | hypoxic | TCGA.3B.A9I3 | normoxic |
| GSM525912 | normoxic | TCGA.K1.A42W | normoxic |
| GSM525913 | normoxic | TCGA.DX.A7EF | hypoxic |
| GSM525914 | normoxic | TCGA.DX.A3LY | normoxic |
| GSM525915 | normoxic | TCGA.VT.A80J | normoxic |
| GSM525916 | normoxic | TCGA.DX.AB37 | normoxic |
| GSM525917 | normoxic | TCGA.VT.A80G | normoxic |
| GSM525918 | normoxic | TCGA.DX.A6B9 | normoxic |
| GSM525919 | hypoxic | TCGA.WK.A8XQ | normoxic |
| GSM525920 | normoxic | TCGA.IF.A4AJ | normoxic |
| GSM525921 | normoxic | TCGA.DX.AB2W | normoxic |
| GSM525922 | normoxic | TCGA.QQ.A5VD | normoxic |
| GSM525923 | normoxic | TCGA.LI.A9QH | normoxic |
| GSM525924 | normoxic | TCGA.SG.A6Z4 | normoxic |
| GSM525925 | normoxic | TCGA.SG.A6Z7 | hypoxic |
| GSM525926 | normoxic | TCGA.IE.A6BZ | normoxic |
| GSM525927 | normoxic | TCGA.DX.A8BX | hypoxic |
| GSM525928 | normoxic | TCGA.HS.A5N9 | hypoxic |
| GSM525929 | normoxic | TCGA.IF.A4AK | normoxic |
| GSM525930 | normoxic | TCGA.DX.A6Z2 | normoxic |
| GSM525931 | normoxic | TCGA.SI.AA8C | hypoxic |
| GSM525932 | normoxic | TCGA.DX.A8BR | normoxic |
| GSM525933 | normoxic | TCGA.UE.A6QU | normoxic |
| GSM525934 | hypoxic | TCGA.X6.A8C6 | normoxic |
| GSM525935 | hypoxic | TCGA.IS.A3K6 | normoxic |
| GSM525936 | normoxic | TCGA.FX.A48G | normoxic |
| GSM525937 | normoxic | TCGA.DX.A1L3 | normoxic |
| GSM525938 | normoxic | TCGA.HB.A3YV | normoxic |
| GSM525939 | normoxic | TCGA.DX.A23V | normoxic |
| GSM525940 | normoxic | TCGA.3B.A9HZ | hypoxic |
| GSM525941 | normoxic | TCGA.DX.A7ER | normoxic |
| GSM525942 | normoxic | TCGA.WK.A8Y0 | normoxic |
| GSM525943 | hypoxic | TCGA.DX.A48K | normoxic |
| GSM525944 | normoxic | TCGA.IF.A3RQ | normoxic |
| GSM525945 | normoxic | TCGA.DX.A7ES | normoxic |
| GSM525946 | normoxic | TCGA.DX.A6YS | normoxic |
| GSM525947 | normoxic | TCGA.MB.A5Y8 | normoxic |
| GSM525948 | normoxic | TCGA.FX.A3TO | normoxic |
| GSM525949 | normoxic | TCGA.DX.A48J | hypoxic |
| GSM525950 | hypoxic | TCGA.DX.A2J1 | normoxic |
| GSM525951 | normoxic | TCGA.DX.AB2Q | normoxic |
| GSM525952 | normoxic | TCGA.DX.A2J0 | normoxic |
| GSM525953 | normoxic | TCGA.DX.AB36 | hypoxic |
| GSM525954 | normoxic | TCGA.WK.A8XT | normoxic |
| GSM525955 | hypoxic | TCGA.QC.AA9N | hypoxic |
| GSM525956 | hypoxic | TCGA.DX.A23T | normoxic |
| GSM525957 | normoxic | TCGA.UE.A6QT | normoxic |
| GSM525958 | normoxic | TCGA.K1.A42X.1 | normoxic |
| GSM525959 | hypoxic | TCGA.DX.A48L | hypoxic |
| GSM525960 | normoxic | TCGA.WK.A8XO | normoxic |
| GSM525961 | normoxic | TCGA.MO.A47P | normoxic |
| GSM525962 | hypoxic | TCGA.DX.A8BP | normoxic |
| GSM525963 | normoxic | TCGA.X6.A7WB | normoxic |
| GSM525964 | normoxic | TCGA.DX.A2IZ | hypoxic |
| GSM525965 | hypoxic | TCGA.X2.A95T | hypoxic |
| GSM525966 | normoxic | TCGA.3B.A9HO | normoxic |
| GSM525967 | normoxic | TCGA.DX.A3U8 | normoxic |
| GSM525968 | normoxic | TCGA.Z4.A8JB | normoxic |
| GSM525969 | normoxic | TCGA.3B.A9HP | normoxic |
| GSM525970 | hypoxic | TCGA.K1.A3PO | normoxic |
| GSM525971 | normoxic | TCGA.K1.A6RT | normoxic |
| GSM525972 | normoxic | TCGA.DX.AB2J | normoxic |
| GSM525973 | normoxic | TCGA.IS.A3K8 | normoxic |
| GSM525974 | normoxic | TCGA.DX.A6B7 | normoxic |
| GSM525975 | normoxic | TCGA.DX.AB2H | normoxic |
| GSM525976 | normoxic | TCGA.DX.A3LS | normoxic |
| GSM525977 | normoxic | TCGA.DX.A3U5 | normoxic |
| GSM525978 | normoxic | TCGA.VT.A80J.1 | normoxic |
| GSM525979 | normoxic | TCGA.DX.A6YV | normoxic |
| GSM525980 | hypoxic | TCGA.DX.A240 | normoxic |
| GSM525981 | normoxic | TCGA.IE.A4EJ | normoxic |
| GSM525982 | hypoxic | TCGA.DX.A6YZ | normoxic |
| GSM525983 | normoxic | TCGA.PC.A5DO | hypoxic |
| GSM525984 | normoxic | TCGA.JV.A75J | hypoxic |
| GSM525985 | normoxic | TCGA.SI.A71P | normoxic |
| GSM525986 | hypoxic | TCGA.X9.A971 | hypoxic |
| GSM525987 | normoxic | TCGA.DX.AB3C | normoxic |
| GSM525988 | normoxic | TCGA.3B.A9HU | normoxic |
| GSM525989 | normoxic | TCGA.DX.A23Z | normoxic |
| GSM525990 | normoxic | TCGA.DX.A8BQ | normoxic |
| GSM525991 | normoxic | TCGA.DX.A8BN | normoxic |
| GSM525992 | normoxic | TCGA.MB.A8JL | normoxic |
| GSM525993 | normoxic | TCGA.IE.A3OV | normoxic |
| GSM525994 | hypoxic | TCGA.QQ.A8VG | normoxic |
| GSM525995 | normoxic | TCGA.N1.A6IA | normoxic |
| GSM525996 | normoxic | TCGA.DX.A3M2 | normoxic |
| GSM525997 | normoxic | TCGA.DX.A7ET | normoxic |
| GSM525998 | normoxic | TCGA.RN.AAAQ | hypoxic |
| GSM525999 | normoxic | TCGA.IW.A3M4 | normoxic |
| GSM526000 | hypoxic | TCGA.QC.A7B5 | normoxic |
| GSM526001 | normoxic | TCGA.DX.A8BO | normoxic |
| GSM526002 | normoxic | TCGA.WK.A8XZ | hypoxic |
| GSM526003 | normoxic | TCGA.X9.A973 | hypoxic |
| GSM526004 | normoxic | TCGA.DX.A6BE | normoxic |
| GSM526005 | hypoxic | TCGA.DX.AB2X | normoxic |
| GSM526006 | hypoxic | TCGA.DX.A7EN | normoxic |
| GSM526007 | normoxic | TCGA.DX.A3LU | normoxic |
| GSM526008 | normoxic | TCGA.KD.A5QS | normoxic |
| GSM526009 | normoxic | TCGA.3B.A9I1 | normoxic |
| GSM526010 | normoxic | TCGA.DX.A6BH | normoxic |
| GSM526011 | normoxic | TCGA.DX.A8BV | normoxic |
| GSM526012 | normoxic | TCGA.SI.A71O.1 | normoxic |
| GSM526013 | normoxic | TCGA.DX.A3M1 | normoxic |
| GSM526014 | hypoxic | TCGA.DX.AB2V | normoxic |
| GSM526015 | normoxic | TCGA.DX.A23U | normoxic |
| GSM526016 | normoxic | TCGA.DX.A1KZ | normoxic |
| GSM526017 | normoxic | TCGA.MB.A5Y9 | normoxic |
| GSM526018 | hypoxic | TCGA.DX.A8BG | normoxic |
| GSM526019 | normoxic | TCGA.DX.A1KU | normoxic |
| GSM526020 | hypoxic | TCGA.K1.A6RV | hypoxic |
| GSM526021 | normoxic | TCGA.DX.A6YT | normoxic |
| GSM526022 | hypoxic | TCGA.HB.A2OT | hypoxic |
| GSM526023 | normoxic | TCGA.3R.A8YX | normoxic |
| GSM526024 | normoxic | TCGA.SI.A71Q | normoxic |
| GSM526025 | normoxic | TCGA.DX.A6YX | hypoxic |
| GSM526026 | normoxic | TCGA.X6.A8C2 | normoxic |
| GSM526027 | normoxic | TCGA.3B.A9HT | hypoxic |
| GSM526028 | hypoxic | TCGA.DX.AB2S | normoxic |
| GSM526029 | hypoxic | TCGA.DX.A48U | normoxic |
| GSM526030 | normoxic | TCGA.DX.A1KX | normoxic |
| GSM526031 | normoxic | TCGA.DX.A3U9 | hypoxic |
| GSM526032 | normoxic | TCGA.PC.A5DK | normoxic |
| GSM526033 | normoxic | TCGA.QQ.A5VC | hypoxic |
| GSM526034 | normoxic | TCGA.3B.A9I0 | normoxic |
| GSM526035 | normoxic | TCGA.IW.A3M5 | normoxic |
| GSM526036 | normoxic | TCGA.PC.A5DL | normoxic |
| GSM526037 | normoxic | TCGA.JV.A5VE | normoxic |
| GSM526038 | hypoxic | TCGA.QQ.A8VB | normoxic |
| GSM526039 | normoxic | TCGA.IE.A4EI | normoxic |
| GSM526040 | hypoxic | TCGA.DX.A6YR | normoxic |
| GSM526041 | normoxic | TCGA.KD.A5QT | normoxic |
| GSM526042 | normoxic | TCGA.HS.A5N7 | hypoxic |
| GSM526043 | normoxic | TCGA.FX.A3NK | normoxic |
| GSM526044 | normoxic | TCGA.DX.A8BL | normoxic |
| GSM526045 | normoxic | TCGA.DX.A3UB | normoxic |
| GSM526046 | normoxic | TCGA.DX.A7EU | normoxic |
| GSM526047 | hypoxic | TCGA.DX.AB32 | normoxic |
| GSM526048 | normoxic | TCGA.WK.A8XX | normoxic |
| GSM526049 | normoxic | TCGA.X6.A8C7 | normoxic |
| GSM526050 | normoxic | TCGA.FX.A2QS | normoxic |
| GSM526051 | hypoxic | TCGA.DX.A7EI | normoxic |
| GSM526052 | normoxic | TCGA.KD.A5QU | normoxic |
| GSM526053 | normoxic | TCGA.3B.A9HX | hypoxic |
| GSM526054 | normoxic | TCGA.QQ.A5VB | normoxic |
| GSM526055 | normoxic | TCGA.K1.A3PN.1 | normoxic |
| GSM526056 | normoxic | TCGA.DX.A3U7 | normoxic |
| GSM526057 | normoxic | TCGA.HS.A5NA | normoxic |
| GSM526058 | normoxic | TCGA.HB.A43Z | normoxic |
| GSM526059 | normoxic | TCGA.DX.A2J4 | normoxic |
| GSM526060 | normoxic | TCGA.IE.A4EH | normoxic |
| GSM526061 | normoxic | TCGA.DX.A8BM | normoxic |
| GSM526062 | hypoxic | TCGA.DX.AB2L | normoxic |
| GSM526063 | normoxic | TCGA.HB.A5W3 | normoxic |
| GSM526064 | normoxic | TCGA.QQ.A8VF | hypoxic |
| GSM526065 | normoxic | TCGA.DX.A8BZ | normoxic |
| GSM526066 | hypoxic | TCGA.3B.A9HJ | normoxic |
| GSM526067 | normoxic | TCGA.DX.A23R | normoxic |
| GSM526068 | normoxic | TCGA.IS.A3K7 | normoxic |
| GSM526069 | normoxic | TCGA.DX.A6BF | normoxic |
| GSM526070 | normoxic |  |  |
| GSM526071 | normoxic |  |  |
| GSM526072 | normoxic |  |  |
| GSM526073 | normoxic |  |  |
| GSM526074 | normoxic |  |  |
| GSM526075 | normoxic |  |  |
| GSM526076 | normoxic |  |  |
| GSM526077 | normoxic |  |  |
| GSM526078 | normoxic |  |  |
| GSM526079 | hypoxic |  |  |
| GSM526080 | normoxic |  |  |
| GSM526081 | normoxic |  |  |
| GSM526082 | normoxic |  |  |
| GSM526083 | normoxic |  |  |
| GSM526084 | normoxic |  |  |
| GSM526085 | normoxic |  |  |
| GSM526086 | hypoxic |  |  |
| GSM526087 | hypoxic |  |  |
| GSM526088 | normoxic |  |  |
| GSM526089 | hypoxic |  |  |
| GSM526090 | hypoxic |  |  |
| GSM526091 | hypoxic |  |  |
| GSM526092 | normoxic |  |  |
| GSM526093 | normoxic |  |  |
| GSM526094 | normoxic |  |  |
| GSM526095 | hypoxic |  |  |
| GSM526096 | normoxic |  |  |
| GSM526097 | normoxic |  |  |
| GSM526098 | normoxic |  |  |
| GSM526099 | normoxic |  |  |
| GSM526100 | normoxic |  |  |
| GSM526101 | normoxic |  |  |
| GSM526102 | normoxic |  |  |
| GSM526103 | normoxic |  |  |
| GSM526104 | hypoxic |  |  |
| GSM526105 | normoxic |  |  |
| GSM526106 | normoxic |  |  |
| GSM526107 | hypoxic |  |  |
| GSM526108 | normoxic |  |  |
| GSM526109 | normoxic |  |  |
| GSM526110 | hypoxic |  |  |
| GSM526111 | normoxic |  |  |
| GSM526112 | normoxic |  |  |
| GSM526113 | normoxic |  |  |
| GSM526114 | normoxic |  |  |
| GSM526115 | hypoxic |  |  |
