# Supplementary material for: Validation of a hypoxia related gene signature in multiple soft tissue sarcoma cohorts
Source: Oncotarget. 2017 Dec 12;9(3):3946–55. doi: 10.18632/oncotarget.23280 (PMC5790513; doi:10.18632/oncotarget.23280)
Supplement: Supplementary file 4 [file oncotarget-09-3946-s004.docx]

**Supplementary Table S11. GO terms and pathways identified by GSEA as enriched with genes down-regulated in high-hypoxia tumors**

| NAME | NES | NOM p-val | FDR q-val |
| --- | --- | --- | --- |
| GO terms | | | |
| GO_HUMORAL_IMMUNE_RESPONSE_MEDIATED_BY_CIRCULATING_IMMUNOGLOBULIN | -2.204378 | 0 | 0 |
| GO_RESPONSE_TO_TYPE_I_INTERFERON | -2.1841977 | 0 | 0 |
| GO_POSITIVE_REGULATION_OF_T_CELL_PROLIFERATION | -2.1557412 | 0 | 0 |
| GO_ACTIVATION_OF_IMMUNE_RESPONSE | -2.032956 | 0 | 0.001475233 |
| GO_REGULATION_OF_T_CELL_PROLIFERATION | -2.0156875 | 0 | 0.001531538 |
| GO_REGULATION_OF_HUMORAL_IMMUNE_RESPONSE | -2.0038564 | 0 | 0.001885471 |
| GO_REGULATION_OF_ACTIVATED_T_CELL_PROLIFERATION | -1.9923016 | 0 | 0.001720825 |
| GO_ADAPTIVE_IMMUNE_RESPONSE | -1.9857382 | 0 | 0.001799748 |
| GO_REGULATION_OF_MACROPHAGE_DERIVED_FOAM_CELL_DIFFERENTIATION | -1.9800603 | 0 | 0.001979961 |
| GO_NEGATIVE_REGULATION_OF_INFLAMMATORY_RESPONSE | -1.9785959 | 0 | 0.001929544 |
| GO_POSITIVE_REGULATION_OF_IMMUNE_RESPONSE | -1.9646024 | 0 | 0.001938514 |
| GO_B_CELL_MEDIATED_IMMUNITY | -1.9517437 | 0 | 0.002330195 |
| GO_LYMPHOCYTE_MEDIATED_IMMUNITY | -1.9430013 | 0 | 0.002683156 |
| GO_ADAPTIVE_IMMUNE_RESPONSE_BASED_ON_SOMATIC_RECOMBINATION_OF_IMMUNE_RECEPTORS_BUILT_FROM_IMMUNOGLOBULIN_SUPERFAMILY_DOMAINS | -1.9261609 | 0 | 0.003884297 |
| GO_REGULATION_OF_INFLAMMATORY_RESPONSE | -1.9255036 | 0 | 0.003854737 |
| GO_REGULATION_OF_CD4_POSITIVE_ALPHA_BETA_T_CELL_ACTIVATION | -1.9117045 | 0 | 0.004805666 |
| GO_REGULATION_OF_INNATE_IMMUNE_RESPONSE | -1.8989525 | 0 | 0.005302365 |
| GO_IMMUNE_EFFECTOR_PROCESS | -1.8866003 | 0 | 0.006399169 |
| GO_INNATE_IMMUNE_RESPONSE | -1.8839512 | 0 | 0.006552783 |
| GO_POSITIVE_REGULATION_OF_INTERFERON_GAMMA_PRODUCTION | -1.88315 | 0 | 0.006519649 |
| GO_REGULATION_OF_INTERFERON_GAMMA_PRODUCTION | -1.8618282 | 0 | 0.009339782 |
| GO_POSITIVE_REGULATION_OF_ACTIVATED_T_CELL_PROLIFERATION | -1.8582528 | 0 | 0.009704702 |
| Pathways | | | |
| BROWNE_INTERFERON_RESPONSIVE_GENES | -2.312647 | 0 | 0 |
| SANA_RESPONSE_TO_IFNG_UP | -2.2968733 | 0 | 0 |
| HECKER_IFNB1_TARGETS | -2.2220864 | 0 | 0 |
| PICCALUGA_ANGIOIMMUNOBLASTIC_LYMPHOMA_UP | -2.2196724 | 0 | 0 |
| MOSERLE_IFNA_RESPONSE | -2.1919088 | 0 | 0 |
| LEE_DIFFERENTIATING_T_LYMPHOCYTE | -2.1860816 | 0 | 0 |
| FULCHER_INFLAMMATORY_RESPONSE_LECTIN_VS_LPS_DN | -2.1796148 | 0 | 0 |
| RADAEVA_RESPONSE_TO_IFNA1_UP | -2.1763792 | 0 | 0 |
| BOSCO_INTERFERON_INDUCED_ANTIVIRAL_MODULE | -2.1699483 | 0 | 0 |
| REACTOME_INTERFERON_ALPHA_BETA_SIGNALING | -2.1273773 | 0 | 0 |
| REACTOME_INNATE_IMMUNE_SYSTEM | -1.9704398 | 0 | 5.13E-04 |
| ZHANG_INTERFERON_RESPONSE | -1.9241569 | 0 | 0.00171187 |
| REACTOME_IMMUNOREGULATORY_INTERACTIONS_BETWEEN_A_LYMPHOID_AND_A_NON_LYMPHOID_CELL | -1.8427926 | 0 | 0.005805555 |
| REACTOME_INTERFERON_SIGNALING | -1.8420798 | 0 | 0.005753698 |
| EINAV_INTERFERON_SIGNATURE_IN_CANCER | -1.8150855 | 0.001862197 | 0.007985849 |
| REACTOME_ANTIGEN_ACTIVATES_B_CELL_RECEPTOR_LEADING_TO_GENERATION_OF_SECOND_MESSENGERS | -1.8134301 | 0 | 0.007971176 |
| REACTOME_CYTOKINE_SIGNALING_IN_IMMUNE_SYSTEM | -1.8057749 | 0 | 0.008678869 |
| HOFFMANN_IMMATURE_TO_MATURE_B_LYMPHOCYTE_UP | -1.7950966 | 0.001855288 | 0.009655289 |
| NES: normalized enrichment score; NOM p-val: nominal P value; FDR q-val: false discovery rate corrected P value | | | |
